# Electronic Structure Tailored Covalent Organic Frameworks for Synergistic Adsorptive–Photocatalytic Gold Recovery from Complex Electronic Waste

**DOI:** 10.34133/research.1012

**Published:** 2025-11-24

**Authors:** Jun Zhang, Lijuan Feng, Jiacheng Zhang, Jianfei Du, Xuewen Cao, Yan Li, Zhanhu Guo, Yihui Yuan, Ning Wang

**Affiliations:** ^1^ School of Marine Sciences, State Key Laboratory of Marine Resource Utilization in South China Sea, Hainan University, Haikou 570228, P. R. China.; ^2^ Department of Mechanical and Civil Engineering, Faculty of Engineering and Environment, Northumbria University, Newcastle Upon Tyne NE1 8ST, UK.

## Abstract

Electronic waste contains gold at concentrations far exceeding natural ores, yet efficient and selective recovery remains challenging. Here, we present a covalent organic framework (COF), tetra-(4-aminophenyl)-porphyrin–4,4′-(thiazolo[5,4-d]thiazol-2,5-diyl)bis(2,5-dimethoxybenzaldehyde)–COF (TAPP-TZ-OMe-COF), in which thiazole and methoxy groups create abundant AuCl_4_^−^ binding sites and modulate the electronic structure via donor–acceptor interactions and p–π conjugation. These features narrow the band gap, enhance charge separation, and accelerate photogenerated electron transfer, enabling synergistic adsorption and visible-light reduction of Au(III) to Au(0). TAPP-TZ-OMe-COF delivers a gold recovery capacity of 4,109 mg g^−1^ and achieves 99.9% recovery from computer processing unit-board leachate with >10^5^-fold selectivity and excellent cycling stability. This molecular-engineering approach offers a generalizable blueprint for integrating adsorption and photocatalysis in COFs, advancing the recovery of gold from complex waste streams for sustainable resource reclamation.

## Introduction

Gold (Au) is a precious metal resource that plays an important role in the global financial system [1]. Attributing to its unique physical properties and chemical stability, gold has also been widely utilized for various practical applications, including electronic communication, catalysis, biomedicine, and aerospace [2–4]. With the rapid development of the electronic industry, the accumulation of global electronic wastes is increasing substantially, and its total amount is predicted to reach 74.7 million tons by 2030 [5,6]. It is notable that the concentration of gold in these electronic wastes is approximately 80 to 100 times that in natural gold ores, and the global electronic waste is estimated to contain 1,568 tons of gold by 2025, presenting as a vast underutilized resource [7]. Furthermore, compared to the mining of gold ores, it is more cost-effective and environmentally friendly to recover gold from electronic waste [8]. Therefore, recovering gold from electronic waste represents a sustainable and efficient way to obtain gold resources.

However, the electronic waste also contains a large number of competitive metal elements, including copper, nickel, and other elements, with much higher contents than gold, which greatly interfere with the recovery of gold [9]. Hence, the development of high-efficiency and selective gold recovery methods is highly essential. Various approaches, including adsorption, chemical reduction, solvent extraction, and electrochemical recovery, have been explored for the recovery of gold from electronic waste [10–12]. However, these techniques still face limitations in terms of recovery efficiency, selectivity, or speed, necessitating further optimization of the electronic waste gold recovery method. The photocatalytic strategy, with its advantages of low energy consumption, ease of operation, high efficiency, and zero harmful emissions, shows high potential for the recovery of gold from electronic waste by reducing soluble AuCl_4_^−^ to insoluble Au(0) [13]. Various photocatalysts have been fabricated for the photocatalytic recovery of gold and to realize high gold recovery efficiencies. Among the developed photocatalysts for gold recovery, covalent organic frameworks (COFs) have emerged as potential candidates for their porous and tunable structure, together with their high chemical stability for practical application in acid environments [14,15].

Benefiting from its modulated and tunable structure, COF can be used as a desirable platform for the rational design of functional photocatalysts. The incorporation of electron donor and acceptor functional units into COFs has been developed into an important strategy for functional regulation of COF photocatalysts, which can contribute to the efficient separation of electron–hole pairs and reduce their recombination, and thus boost photocatalytic efficiency [16]. Except for the regulation of the photocatalyst electronic structure, the synergy of adsorption and photocatalysis has also been proven to greatly enhance the recovery ability for metal ions, which is due to the preadsorption of metal ions that can increase the partial metal ion concentration, lower the activation energy, and enhance reaction selectivity, thereby improving the metal ion recovery performance. Hence, the introduction of abundant gold adsorption sites and the optimization of the electronic structure can be a potential approach for boosting the gold recovery efficiency.

**Fig. 1.**
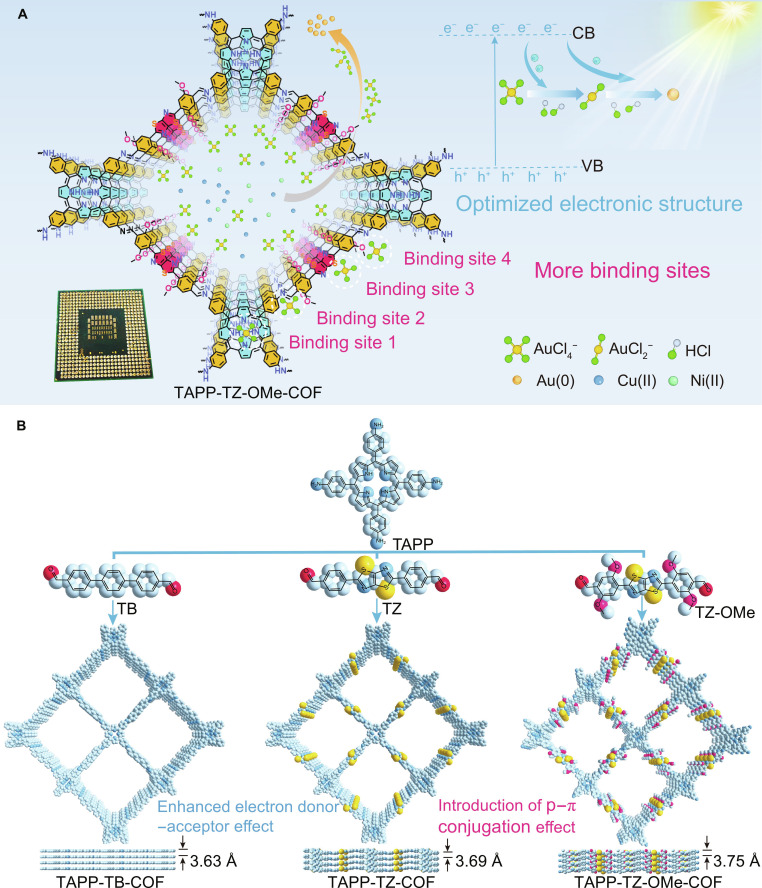
Schematic of the strategy to boost gold recovery performance of COFs. (A) Schematic for boosting gold recovery efficiency by optimizing the electronic structure and increasing gold binding sites in COFs. (B) Synthesis of COFs with modulated structural characteristics to enhance gold recovery efficiency.

In this study, we propose a new strategy for optimizing the gold recovery performance of COF photocatalysts through the regulation of the electronic structure and the increase in binding sites (Fig. 1A). Based on the excellent electronic properties of porphyrin, the organic ligand tetra-(4-aminophenyl)-porphyrin (TAPP) is used as electron donor, and the organic ligands (1,1′:4′,1″-terphenyl)-4,4″-dicarbaldehyde (TB), 4,4′-(thiazolo[5,4-d]thiazol-2,5-diyl)dibenzaldehyde (TZ), and 4,4′-(thiazolo[5,4-d]thiazol-2,5-diyl)bis(2,5-dimethoxybenzaldehyde) (TZ-OMe) are used as electron acceptors to synthesize the COF materials TAPP-TB-COF, TAPP-TZ-COF, and TAPP-TZ-OMe-COF, respectively. Furthermore, the electron donor–acceptor effect between the porphyrin group and the thiazole group together with the p–π conjugation effect between the methoxy group and the benzene ring in TAPP-TZ-OMe-COF enhance the separation and reduce the recombination of electron–hole pairs and promote the rapid transport of photogenerated electrons [17,18]. Consequently, the gold recovery capacity of TAPP-TZ-OMe-COF is greatly improved compared to TAPP-TB-COF and TAPP-TZ-COF. Under light irradiation, an outstanding gold recovery capacity of 4,109 mg g^−1^ is achieved by TAPP-TZ-OMe-COF. More importantly, TAPP-TZ-OMe-COF can recover gold from waste computer processing unit (CPU) with 99.9% efficiency together with high selectivity against coexisting metal ions, placing it as one of the best-performing photocatalysts for gold recovery.

## Results

### Synthesis and characterization of COFs

**Fig. 2.**
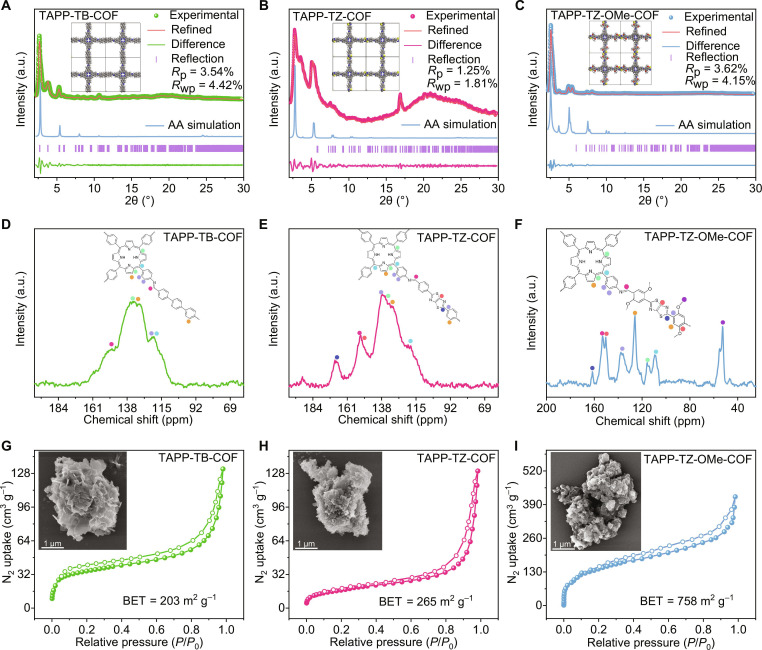
Characterization of the as-synthesized COFs. Experimental PXRD patterns and simulated PXRD patterns of the eclipsed AA stacking model of (A) TAPP-TB-COF, (B) TAPP-TZ-COF, and (C) TAPP-TZ-OMe-COF. ^13^C CP-MAS NMR spectra of (D) TAPP-TB-COF, (E) TAPP-TZ-COF, and (F) TAPP-TZ-OMe-COF. N_2_ adsorption–desorption isotherms of (G) TAPP-TB-COF, (H) TAPP-TZ-COF, and (I) TAPP-TZ-OMe-COF at 77 K. The corresponding SEM images are also shown.

Based on the modulated structure of COFs, the optimization of electronic structure and the introduction of more adsorption sites would be promising strategies to boost the photocatalytic performance of COF photocatalysts. To develop COFs with superior photocatalytic gold recovery efficiency, organic ligand TAPP that contains electron-donating porphyrin groups was selected as electron donor. The electron-accepting ligands TB, TZ, and TZ-OMe were used as electron acceptors to synthesize a series of COFs, namely, TAPP-TB-COF, TAPP-TZ-COF, and TAPP-TZ-OMe-COF, through a Schiff base condensation reaction (Fig. 1B). The crystallinity of these COFs was characterized by powder x-ray diffraction (PXRD) analysis, which confirmed that all 3 COFs exhibited a highly crystalline structure (Fig. 2A to C). The geometric structure analysis showed that the structures of these 3 COFs fitted better with the AA stacking model, and their structure all belonged to the triclinic *P*1 space group (Figs. S1 to S3 and Tables S1 to S3).

The successful synthesis of the COFs was further analyzed. Solid-state ^13^C nuclear magnetic resonance (NMR) analysis confirmed the presence of characteristic carbon signals of the -C=N- group at around 153 parts per million (ppm) for all 3 COFs, proving their synthesis through the Schiff base condensation reaction (Fig. 2D to F). Except for TAPP-TB-COF, the characteristic carbon signals at 150 ppm [19], corresponding to the introduced thiazole groups, were observed in the NMR spectra of TAPP-TZ-COF and TAPP-TZ-OMe-COF. Additionally, the characteristic carbon signal of the -OCH_3_ group was only observed in the spectrum of TAPP-TZ-OMe-COF at around 50 ppm, confirming the successful incorporation of the -OCH_3_ group [20]. The Fourier transform infrared spectrum analysis showed that all 3 COFs exhibited a characteristic peak at 1,610 cm^−1^, corresponding to the stretching vibration of -C=N-, further validating the synthesis of these COFs via Schiff base condensation (Fig. S4). Unlike the spectrum of TAPP-TB-COF, the characteristic peaks for the thiazole group at 649 cm^−1^, corresponding to -C-S-C, were observed in the spectra of TAPP-TZ-COF and TAPP-TZ-OMe-COF. Moreover, the specific peak at 1,218 cm^−1^ for the -OCH_3_ group was also observed in TAPP-TZ-OMe-COF [21,22]. The x-ray photoelectron spectroscopy (XPS) analysis showed that the N 1*s* peaks for TAPP-TZ-OMe-COF, TAPP-TZ-COF, and TAPP-TB-COF appeared at binding energies of 399.10, 399.34, and 399.50 eV, respectively, indicating the formation of imine bonds via the Schiff base condensation reaction (Fig. S5) [23]. The signals of the protonated N atom in the form of -NH- in the porphyrin group were observed at binding energies of 397.48, 397.68, and 397.91 eV for the 3 COFs, respectively. Although these N atoms were derived from the same chemical group, they exhibited different binding energies, which was attributed to the different electronic structures of these 3 COFs. This variation correlates with the donor–acceptor interactions within the frameworks. The porphyrin units act as electron donors, while the TB, TZ, and TZ-OMe linkers provide progressively stronger acceptor effects. The enhanced charge delocalization and p–π conjugation in the TZ-OMe linker increase electron density around nitrogen, leading to the observed negative shift in binding energy. The characterizations of the synthesized COFs were further investigated. N_2_ adsorption–desorption isotherms revealed that the Brunauer–Emmett–Teller (BET) surface areas for TAPP-TB-COF, TAPP-TZ-COF, and TAPP-TZ-OMe-COF were 203, 265, and 758 m^2^ g^−1^, respectively (Fig. 2G to I). The substantially increased BET surface area of TAPP-TZ-OMe-COF can be attributed to the introduction of methoxy groups, which expand the interlayer spacing of the COF, thereby increasing their surface area, which can further benefit the interaction of gold with the materials. The analysis of the pore size distributions of these 3 COFs also proved that TAPP-TZ-OMe-COF exhibited bigger pore size, which was consistent with the interlayer distance of these COFs (Fig. 1B and Fig. S6). The scanning electron microscopy (SEM) observations showed that all 3 COFs existed in a 2-dimensional nanosheet structure, which can also benefit the interaction of gold with the materials (Fig. 2G to I). Additionally, the results of thermogravimetric analysis (TGA) showed that no substantial weight loss was observed up to 300 °C under a nitrogen atmosphere, indicating that the COFs possessed excellent thermal stability (Fig. S7). TAPP-TZ-OMe-COF exhibited outstanding wettability, with the contact angle decreasing to 37.3° within 0.1 s (Fig. S8). This pronounced hydrophilic behavior makes TAPP-TZ-OMe-COF an ideal candidate for the efficient separation of AuCl_4_^−^ from aqueous solutions.

### Gold recovery performance of COFs

To evaluate the optimization environment for gold recovery by COFs, the influence of pH on the gold recovery performance was analyzed and the most dominant valence of gold [Au(III)] was used as the target. The result showed that, within the pH range from 1 to 7, the increase in pH led to a gradual decrease in gold recovery ability, which was due to the influence of pH on the gold ion species and the state of the binding sites (Fig. S9). With pH below 4, the gold existed in the form of AuCl_4_^−^, and the functional binding sites, including the N atom from porphyrin groups, the N atom from the imine bonds, the S atom from thiazole groups, and the O atom from methoxy groups, were electropositive due to protonation. The electrostatic interaction facilitated the enrichment of gold, which further benefited the photocatalytic process by increasing the local concentration of metal ions and lowering the activation energy. As pH values increase, AuCl_4_^−^ underwent a hydrolysis process to form gold ion complexes, including Au(OH)Cl_3_^−^, Au(OH)_2_Cl_2_^−^, Au(OH)_3_Cl^−^, and Au(OH)_4_^−^, and the protonation level of the binding sites was decreased [24]. This trend reflects the synergistic influence of pH-dependent Au species and the protonation state of the active sites, jointly governing the electrostatic adsorption process. Thus, the gold recovery ability decreased along with the further increase in pH values.

**Fig. 3.**
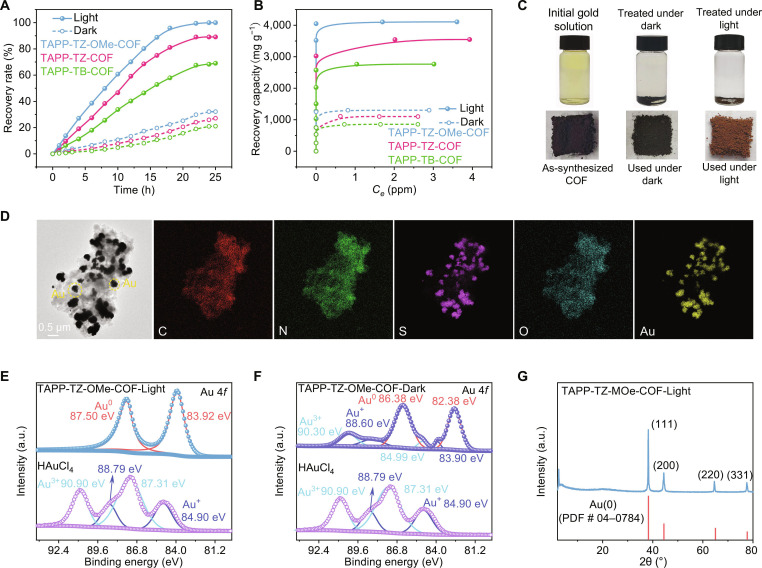
Gold recovery performance and characterizations of COFs after being used for gold recovery. (A) Gold recovery kinetics of COFs with gold concentration of 16 ppm and dosage of 250 mg l^−1^. (B) Gold recovery isotherms of COFs. (C) Morphologies of the gold solution and COFs before and after being used for gold recovery under dark and under light, respectively. (D) FE-TEM image and corresponding EDS elemental mapping of TAPP-TZ-OMe-COF after being used for gold recovery under light irradiation. (E) XPS spectrum of Au 4*f* before and after being recovered by TAPP-TZ-OMe-COF under light irradiation. (F) XPS spectrum of Au 4*f* before and after being recovered by TAPP-TZ-OMe-COF under dark irradiation. (G) PXRD spectra of TAPP-TZ-OMe-COF after being used for gold recovery under light irradiation.

To further investigate the gold recovery behavior of the COFs, the recovery kinetics was determined under dark and light irradiation with a light density of 1 sun (1 kW m^−2^) (Fig. 3A). The results showed that, at a dosage of 250 mg l^−1^, TAPP-TZ-OMe-COF can recover 24% and 95% of the gold within 18 h under dark and light irradiation, respectively. The higher gold recovery efficiency under light irradiation was attributed to the role of photocatalytic gold recovery. Compared with TAPP-TZ-OMe-COF, TAPP-TB-COF and TAPP-TZ-COF exhibited both lower recovery efficiency and slower recovery rate. The gold recovery isotherms have also been determined (Fig. 3B). The results showed that, under dark conditions, the gold recovery capacities of TAPP-TB-COF, TAPP-TZ-COF, and TAPP-TZ-OMe-COF were 853, 1,100, and 1,301 mg g^−1^, respectively, within 24 h. The irradiation of light greatly boosted the gold recovery performance, and TAPP-TB-COF, TAPP-TZ-COF, and TAPP-TZ-OMe-COF realized gold recovery capacities of 2,766, 3,548, and 4,109 mg g^−1^, which were 3.24, 3.23, and 3.16 times those under dark conditions, respectively, proving the great ability of photocatalysis in boosting gold recovery. To elucidate the role of preadsorption in photocatalytic gold recovery, a control experiment was conducted in which AuCl_4_^−^ adsorption on TAPP-TZ-OMe-COF was allowed to reach equilibrium in the dark before light irradiation. The preadsorbed sample exhibited a substantially higher gold recovery rate than direct illumination (Fig. S10), confirming that surface enrichment of gold enhanced electron transfer and photocatalytic reduction efficiency. These results indicated that the optimization of the electronic structure and the incorporation of more binding sites both benefited the gold recovery performance, including the recovery capacity and rate. The gold recovery capacity of TAPP-TZ-OMe-COF is among the best-performing COFs, proving the high efficiency of the strategies used in this study (Table S4).

The characteristics of the COFs before and after gold recovery were systematically investigated. Upon exposure to light irradiation during the gold recovery process, TAPP-TZ-OMe-COF exhibited a distinct color transition from black to yellow, concurrently with the gold solution changing from yellow to colorless, indicating the effective gold uptake and reduction (Fig. 3C and Fig. S11). Field emission transmission electron microscopy (FE-TEM) coupled with energy-dispersive x-ray spectroscopy (EDS) revealed a substantially higher density of gold nanoparticles deposited on TAPP-TZ-OMe-COF compared to TAPP-TB-COF and TAPP-TZ-COF, confirming its superior recovery performance (Fig. 3D and Fig. S12). The XPS analysis demonstrated that, under light irradiation, gold on the COFs predominantly existed in the metallic Au(0) state, which was attributed to the photocatalytic reduction of Au(III) (Fig. 3E and Fig. S13). In contrast, for COFs treated under dark conditions, gold existed in multiple valences, including Au(III), Au(I), and Au(0), with Au(0) as the dominant state, indicating the occurrence of the chemical reduction of AuCl_4_^−^ under dark conditions (Fig. 3F and Fig. S14) [25,26]. The PXRD patterns corroborated these findings, exhibiting sharp characteristic peaks corresponding to crystalline metallic gold (PDF # 04-0784) (Fig. 3G and Fig. S15) [27,28]. Collectively, these results elucidated that the combination of adsorption, chemical reduction, and photocatalytic reduction mechanisms under light irradiation substantially enhanced the gold recovery efficiency of TAPP-TZ-OMe-COF.

**Fig. 4.**
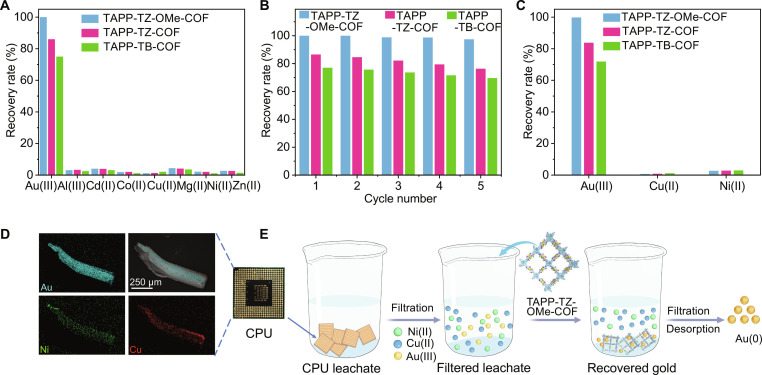
Gold recovery performance of COFs from CPU leachate. (A) Gold recovery selectivity of COFs in simulated solution containing various metal ions under light irradiation. (B) Reusability of COFs for gold recovery under light irradiation. (C) Recovery of gold from CPU board leachate by COFs. (D) SEM image and corresponding EDS elemental mapping of CPU board. (E) The processes for gold recovery from waste CPU board by TAPP-TZ-OMe-COF.

The presence of coexisting metal ions in electronic waste poses a substantial challenge for selective gold recovery and material reusability. To simulate the leachate composition of electronic waste, a mixture containing metal ions, including Au(III), Al(III), Cd(II), Co(II), Cu(II), Mg(II), Zn(II), and Ni(II), each at a concentration of 100 ppm, was prepared. Despite the complex interference from these competing metal ions, TAPP-TZ-OMe-COF demonstrated outstanding selectivity for gold, achieving a gold recovery efficiency exceeding 99% (Fig. 4A). This indicates that TAPP-TZ-OMe-COF effectively discriminates gold ions from other metal ions. For reusability, an elution solution composed of 0.25 M thiourea and 0.25 M hydrochloric acid was used, achieving an impressive gold elution efficiency of 97% within 8 h, thus confirming the effectiveness of this elution method (Fig. S16). After 4 cycles of gold recovery and elution, TAPP-TZ-OMe-COF maintained a gold recovery efficiency greater than 99%, demonstrating exceptional reusability and stability (Fig. 4B).

Given its exceptional selectivity and reusability, the practical applicability of TAPP-TZ-OMe-COF for gold recovery from real electronic waste was further investigated by using a CPU board (Fig. 4C to E). The metal elements on the CPU board were leached using aqua regia, and the resulting leachate contained Cu(II), Ni(II), and Au(III) at concentrations of 832.2, 162.8, and 10.54 ppm, respectively. A 10-mg sample of TAPP-TZ-OMe-COF was added to 50 ml of the CPU board leachate and subjected to gold recovery under light irradiation for 4 h. The results demonstrated that 99.9% of the gold can be successfully recovered from the leachate, significantly outperforming Cu and Ni, with their recovery efficiencies being only 0.69% and 2.69%, respectively. The gold recovery selectivity of TAPP-TZ-OMe-COF was quantified by calculating the distribution coefficients (*k*_d_), which were found to be 5 × 10^6^ ml g^−1^ for Au(III), 34.7 ml g^−1^ for Cu(II), and 138.22 ml g^−1^ for Ni(II). These results revealed that the gold recovery selectivity of TAPP-TZ-OMe-COF was approximately 1.44 × 10^5^ times greater than that for Cu(II) and 3.62 × 10^4^ times greater than that for Ni(II). TAPP-TZ-OMe-COF exhibited excellent selectivity for AuCl_4_^–^ in CPU and competitive ion solutions, which can be attributed to the synergistic effects of its tailored adsorption sites. According to the hard–soft acid–base principle, the soft Au(III) centers in AuCl_4_^−^ preferentially interact with the soft Lewis base sites (S, N, and O) within TAPP-TZ-OMe-COF. The electron-donating methoxy and thiazole groups increase the softness and polarizability of these donor atoms, strengthening the soft–soft coordination. Specifically, the methoxy group enhances the electron density of the thiazole units, rendering the sulfur and oxygen atoms softer and more electron-rich, which facilitates their coordination with the soft acid Au(III) centers. In contrast, competing ions such as Cu(II), Ni(II), and Al(III), classified as harder acids, exhibited weaker affinity, ensuring outstanding recognition of AuCl_4_^−^. Collectively, these findings highlight the strong practical potential of TAPP-TZ-OMe-COF for gold recovery from actual electronic waste.

### Mechanism for gold recovery under dark conditions

**Fig. 5.**
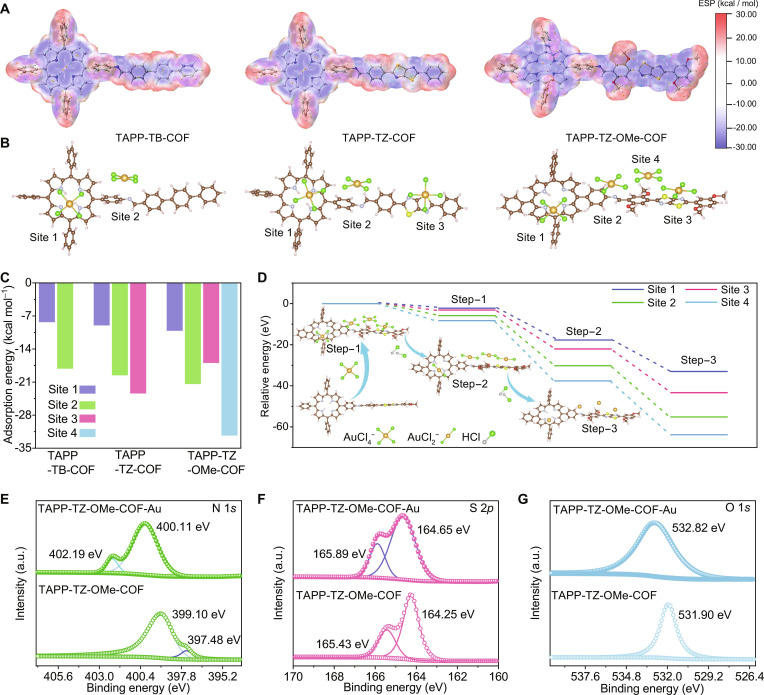
Gold recovery mechanism of COFs under dark conditions. (A) Calculated ESP map of the COFs. The blue color represents negative potential and the red color represents positive potential. (B) Structure of AuCl_4_^−^ bound by specific binding sites in COFs. (C) Comparison of the binding energies of specific binding sites in COFs for AuCl_4_^−^. (D) DFT-calculated energy differences during the gold recovery process of TAPP-TZ-OMe-COF. XPS spectra of (E) N 1*s*, (F) S 2*p*, and (G) O 1*s* in TAPP-TZ-OMe-COF after being used in gold recovery under dark conditions.

The analysis of the gold recovery process revealed that the COFs primarily recover gold through 2 distinct mechanisms: adsorption–reduction under dark conditions and photocatalysis under light irradiation. Thus, the mechanisms for these 2 ways were thoroughly examined. To explore the chemical adsorption mechanism of AuCl_4_^−^ by COFs, density functional theory (DFT) calculations were employed. The calculations of the electrostatic potential (ESP) and binding energies at specific sites for AuCl_4_^−^ demonstrated that TAPP-TZ-OMe-COF provided 4 potential binding sites for AuCl_4_^−^, which included the N atom from the porphyrin group, the N atom from the imine bond, the S atom from the thiazole group, and the O atom from the methoxy group (Fig. 5A and B). In contrast, TAPP-TB-COF and TAPP-TZ-COF only possessed 2 and 3 binding sites, respectively, which is a key factor contributing to the higher gold adsorption ability of TAPP-TZ-OMe-COF. Additionally, the introduction of thiazole and methoxy groups altered the electronic structure of the COFs, thereby influencing the binding energies at these sites for AuCl_4_^−^. The calculated binding energies of the porphyrin group, thiazole group, imine bond, and methoxy group for AuCl_4_^−^ in TAPP-TZ-OMe-COF were found to be −10.14, −16.96, −21.41, and −32.33 kcal mol^−1^, respectively. Among these binding sites, the methoxy group exhibited the highest binding energy, and most of the binding sites in TAPP-TZ-OMe-COF showed higher binding energies than corresponding sites in TAPP-TZ-COF and TAPP-TB-COF (Fig. 5C). Furthermore, DFT calculations were performed to compare the binding energies of Au(III), Cu(II), and Ni(II) at different adsorption sites of TAPP-TZ-OMe-COF. The results showed that all sites exhibited substantially stronger binding toward Au(III) than toward Cu(II) or Ni(II) (Fig. S17). This finding is consistent with the experimentally observed selectivity, indicating that the stronger coordination of Au(III) accounts for its high distribution coefficient. The presence of more binding sites and the higher binding energy at each site contributed to the superior gold recovery ability of TAPP-TZ-OMe-COF.

To reveal the generation mechanism of gold nanoparticles under dark conditions, the adsorption–reduction mechanism was analyzed by DFT calculations (Fig. 5D). The results showed that the adsorption sites in TAPP-TZ-OMe-COF firstly capture AuCl_4_^−^ from the aqueous solution, proven by the DFT-calculated energy differences (Δ*E*_1_) of −2.27, −3.13, −5.85, and −8.31 eV (Step 1). Subsequently, the protonated binding sites provide protons to interact with Cl^−^ in AuCl_4_^−^ for forming HCl and Au(III), which was chemically reduced to form Au(I) by the binding sites, confirmed by the energy differences (Δ*E*_2_) of each binding site of −15.45, −18.94, −24.35, and −29.30 eV (Step 2). Finally, Au(I) in AuCl_2_^−^ was further chemically reduced to Au(0) by the binding sites, proven by the energy differences (Δ*E*_3_) of each binding site of −15.32, −21.26, −24.97, and −26.22 eV, respectively, and Cl^−^ formed HCl by interacting with protons (Step 3). Notably, the methoxy and thiazole sites exhibit particularly favorable energy profiles in Steps 2 and 3, primarily due to their strong electronic effects and partially to structural features. The methoxy group enhances local electron density via p–π conjugation, facilitating electron transfer for Au(III) reduction. The XPS analysis further confirmed the function of the binding sites in enriching gold. The results indicated that, after being used in gold recovery under dark conditions, the binding energies of N 1*s*, S 2*p*, and O 1*s* were all increased, indicating the functions of the binding sites in providing electrons for the chemical reduction of Au(III) to Au(0) (Fig. 5E to G and Figs. S18 and S19) [29,30]. In addition, the electron paramagnetic resonance signal for oxygen in TAPP-TZ-OMe-COF markedly decreased after AuCl_4_^–^ adsorption, suggesting the interaction between the oxygen sites and Au(III) (Fig. S20).

### Mechanism for gold recovery by photocatalysis

**Fig. 6.**
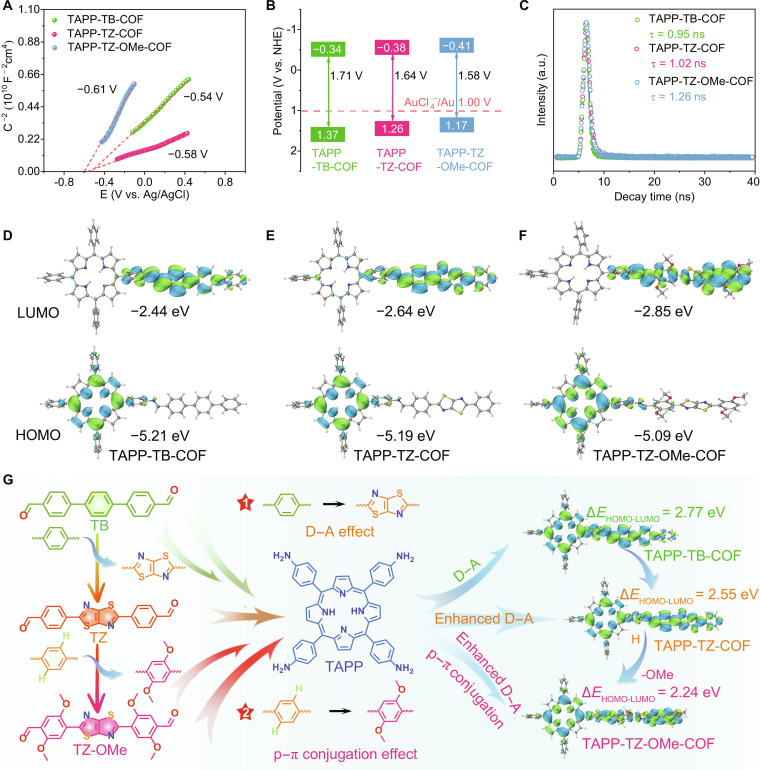
Photocatalytic recovery mechanism of gold by COFs. (A) Mott–Schottky plot of COFs. (B) Energy band positions of COFs. (C) Time-resolved photoluminescence spectra of COFs. (D) Energy level diagram of (D) TAPP-TB-COF, (E) TAPP-TZ-COF, and (F) TAPP-TZ-OMe-COF. (G) Optimization of band gap and enhancement of photocatalytic gold recovery efficiency in COFs via the donor–acceptor effect and the p–π conjugation effect.

Photocatalysis has been established as the primary mechanism for the efficient recovery of gold from electronic waste. Therefore, the characteristics and mechanisms underlying the photocatalytic gold recovery ability of the COFs were thoroughly investigated. Ultraviolet–visible diffuse reflectance spectroscopy (UV/Vis DRS) analysis of the COFs demonstrated that all 3 COFs exhibited broad light absorption across both the UV and Vis regions. Notably, TAPP-TZ-OMe-COF showed the highest light absorption ability (Fig. S21). Tauc plots derived from the UV/Vis DRS data revealed that the optical band gaps (*E*_g_) for TAPP-TZ-OMe-COF, TAPP-TZ-COF, and TAPP-TB-COF were 1.58, 1.64, and 1.71 eV, respectively (Fig. S22). These results indicated that the incorporation of thiazole and methoxy groups progressively enhanced the light absorption properties and reduced the band gaps of the COFs. Mott–Schottky analysis confirmed that all 3 COFs, TAPP-TZ-OMe-COF, TAPP-TZ-COF, and TAPP-TB-COF, are n-type semiconductors [31,32], with flat band potentials of −0.61, −0.58, and −0.54 V (relative to the Ag/AgCl reference electrode), corresponding to conduction band (CB) energy levels of −0.41, −0.38, and −0.34 V (relative to NHE), respectively (Fig. 6A). The n-type nature of these COFs indicates that photogenerated electrons in the CB serve as active reducing agents, facilitating the transfer of electrons to AuCl_4_^−^ and promoting the reduction of Au(III) to Au(0). Further electrochemical analysis provided the valence band (VB) potentials of TAPP-TZ-OMe-COF, TAPP-TZ-COF, and TAPP-TB-COF as 1.17V, 1.26, and 1.42 V, respectively (Fig. 6B). These VB potentials are all suitable for the photocatalytic reduction of Au(III) to Au(0) (1.002 V vs. NHE), while the corresponding photogenerated holes possess sufficient oxidation potential to oxidize water molecules to O_2_ (0.85 V vs. NHE), thereby maintaining charge balance within the system [33]. To elucidate the dependence of photocatalytic performance on light parameters, TAPP-TZ-OMe-COF was evaluated under monochromatic illumination at 365, 630, 750, and 850 nm. The highest gold recovery capacity was achieved at 630 nm, which corresponds to the main absorption band of TAPP-TZ-OMe-COF, while the lower recovery values observed at 365, 750, and 850 nm indicate reduced activity at wavelengths outside the principal absorption region. In addition, under simulated sunlight with different intensities of 0.5, 1.0, 1.5, and 2.0 kw m^−2^, the gold recovery capacity increased with increasing light intensity (Fig. S23). This result indicates that stronger illumination generates more photoinduced charge carriers, thereby accelerating the reduction of AuCl_4_^−^ to metallic Au(0). These findings confirm that the photocatalytic gold recovery process of the COFs is dependent on both wavelength and light intensity.

Photoelectrochemical measurements were conducted to investigate the photoelectric properties of the COFs. Under periodic light switching, all COFs exhibited anodic photocurrents, with TAPP-TZ-OMe-COF displaying the highest photocurrent density, indicative of its superior electron separation and transport abilities (Fig. S24) [34–36]. Electrochemical impedance spectroscopy analysis further revealed that TAPP-TZ-OMe-COF displayed a smaller semicircle in the Nyquist plot, suggesting its lower charge transfer resistance (Fig. S25). The photoluminescence (PL) spectra of the COFs showed that TAPP-TZ-OMe-COF exhibited the lowest PL intensity and longest fluorescence lifetime, revealing that TAPP-TZ-OMe-COF possessed a lower electron–hole pair recombination rate after photoexcitation (Fig. 6C and Fig. S26) [37,38]. The electronic structures of the COFs were investigated using DFT calculations to determine the energy levels of the frontier orbitals (highest occupied molecular orbital [HOMO] and lowest unoccupied molecular orbital [LUMO]). The calculations revealed that the HOMOs of TAPP-TZ-OMe-COF, TAPP-TZ-COF, and TAPP-TB-COF were positioned at −5.09, −5.19, and −5.21 eV, respectively, while the LUMOs were located at −2.85, −2.64, and −2.44 eV, respectively (Fig. 6D to F). The electronic structures of the COFs exhibited typical donor–acceptor configurations, with the TAPP group serving as the electron donor and TB, TZ, and TZ-OMe functioning as electron acceptors [39–41]. The introduction of the thiazole and methoxy groups led to an elevation in the HOMO energy levels of the COFs, which, in turn, enhanced the ability of these materials to release photogenerated electrons. Additionally, the HOMO–LUMO band gaps of the 3 COFs were 2.24, 2.55, and 2.77 eV, respectively, indicating that the synergistic effect of the donor–acceptor interaction induced by the thiazole group and the p–π conjugation effect generated by the methoxy group reduced the band gap, optimized the electron distribution, and promoted electron transfer. Time-dependent DFT calculations were also performed, revealing charge-transfer excitations from the porphyrin donor units to the thiazole or methoxy-containing acceptor sites (Fig. S27). These findings suggest that the introduction of the thiazole and methoxy groups effectively improves electron separation and transport while suppressing recombination of electron–hole pairs, thus enhancing the efficiency of photocatalytic gold recovery by providing photogenerated electrons more efficiently (Fig. 6G).

## Conclusion

The rational design of COFs has demonstrated substantial potential in enhancing gold recovery from electronic waste. In this study, strategies including the optimization of the electronic structure and the increase of the binding sites of COFs were integrated, facilitating an improved gold recovery performance through the gradual introduction of thiazole and methoxy groups. The presence of multiple adsorption sites, including N atoms from porphyrin and imine groups, sulfur atoms from thiazole groups, and oxygen atoms from methoxy groups, contributes to enhanced binding for AuCl_4_^−^, with TAPP-TZ-OMe-COF exhibiting the highest gold recovery efficiency under dark conditions. The preadsorption of AuCl_4_^−^ further increased the concentration of gold ions at the surface and lowered the activation energy for photocatalytic gold recovery by reducing soluble Au(III) to insoluble Au(0), thus improving the overall recovery efficiency. The thiazole group, being an electron-withdrawing entity, optimized the electronic structure of the COFs through an electron donor–acceptor effect with the porphyrin thiazole group. Meanwhile, the methoxy group further enhanced the electronic properties of the COF through the p–π conjugation between the oxygen atom’s p-orbital and the π-orbital of the benzene ring. Together, the electron donor–acceptor and the p–π conjugation effects optimized the electronic structure, thereby improving electron separation and transport, as well as suppressing the recombination of electron–hole pairs, thus providing more photogenerated electrons for efficient photocatalytic gold recovery. As a result, the optimized TAPP-TZ-OMe-COF photocatalyst achieved an exceptional gold recovery capacity of 4,109 mg g^−1^, making it one of the most efficient photocatalysts for gold recovery. Additionally, owing to the inherent stability of COFs, TAPP-TZ-OMe-COF demonstrated excellent reusability. In real-world tests using CPU leachate, TAPP-TZ-OMe-COF successfully recovered 99.9% of the gold, even with the presence of over 90 times the concentration of interfering ions, highlighting its strong practical application potential. Combining the strategies of optimized electronic structure and increased adsorption sites, this study designed a new COF with outstanding gold recovery performance. The findings of this study would also guide the rational design of efficiently photocatalysts.

## Materials and Methods

### Materials

All reagents were obtained from commercial sources and used as received. TAPP, TB, TZ, and TZ-OMe were purchased from Jilin Zhongke Yanshen Technology Co., Ltd. Tetrachloroauric acid trihydrate (HAuCl_4_·3H₂O), *o*-dichlorobenzene (*o*-DCB), and *n*-butanol (*n*-BuOH) were obtained from Shanghai Aladdin Biochemical Technology Co., Ltd. *N*,*N*-dimethylformamide (DMF), tetrahydrofuran (THF), hydrochloric acid (HCl), nitric acid (HNO_3_), acetic acid (AcOH), sodium hydroxide (NaOH), and thiourea were purchased from Shanghai Macklin Biochemical Technology Co., Ltd. and Xilong Scientific Co., Ltd.

### Synthesis of COFs

TAPP (0.02 mmol, 13.78 mg) and TB (0.04 mmol, 11.69 mg) were dispersed in the mixture of 1,2-dichlorobenzene (0.8 ml), *n*-butanol (0.2 ml), and acetic acid (6 M, 0.1 ml), placed in a Pyrex tube (10 ml), and degassed through 3 freeze–pump–thaw cycles to synthesize TAPP-TB-COF. TAPP (0.02 mmol, 13.78 mg) and TZ (0.04 mmol, 14.75 mg) were dispersed in the mixture of 1,2-dichlorobenzene (0.8 ml), *n*-butanol (0.2 ml), and acetic acid (6 M, 0.1 ml), placed in a Pyrex tube (10 ml), and degassed through 3 freeze–pump–thaw cycles to synthesize TAPP-TZ-COF. TAPP (0.02 mmol, 13.78 mg) and TZ-OMe (0.04 mmol, 19.83 mg) were dispersed in the mixture of 1,2-dichlorobenzene (1 ml) and acetic acid (6 M, 0.1 ml), placed in a Pyrex tube (10 ml), and degassed through 3 freeze–pump–thaw cycles to synthesize TAPP-TZ-OMe-COF. The tube was flame-sealed and heated at 120 °C for 5 days. The precipitate was collected by centrifugation, washed 3 times with THF, washed twice with DMF, and Soxhlet-extracted with THF for 1 day. The powder was collected and vacuum-dried at 80 °C overnight to obtain TAPP-TB-COF, TAPP-TZ-COF, and TAPP-TZ-OMe-COF with a yield of 65.0%, 76.2%, and 75.3%, respectively.

### Optimal pH for gold recovery

To determine the optimal pH for gold adsorption, 100 ppm gold solution was prepared, and the pH of the solution was adjusted to a final pH of 1.0 to 7.0 using 2 M NaOH and 1 M HNO_3_. Then, 5 mg of material was dispersed into 20 ml of gold solution at different pH values, and the mixture was stirred under light for 4 h. A 100-μl sample of the solution was taken and filtered through a 0.22-μm filter membrane. The remaining concentration of gold was measured using inductively coupled plasma–optical emission spectroscopy (ICP-OES). The recovery rate (%) was calculated using the following formula:

$$\text{Recovery rate}=\frac{\left(C_{0}-C_{e}\right)}{C_{0}}\times 100\%$$ (1)

where *C*₀ (mg l^−1^) is the initial gold concentration and *C*_e_ (mg l^−1^) is the equilibrium concentration of gold.

### Batch gold recovery performance assay

Gold ion aqueous solutions of different concentrations (1 to 20 ppm) were prepared using HAuCl_4_·3H₂O and stored in the dark at 4 °C for use. The Au(III) solutions were wrapped with aluminum foil to prevent exposure to light during the recovery performance assay under dark conditions. COF (2 mg) was immersed into the above solutions (500 ml) of different concentrations at room temperature, stirring for 24 h either in the dark or under light. The mixture was then filtered using a 0.22-μm membrane filter. The concentration of metals in the resulting filtrate was measured using ICP-OES. The recovery capacity (mg g^−1^) was calculated using the following formula:

$$\text{Recovery capacity}=\frac{\left(C_{0}-C_{e}\right)}{m}\times V$$ (2)

where *C*₀ (mg l^−1^) is the initial gold concentration, *C*_e_ (mg l^−1^) is the equilibrium gold concentration, *V* (l) is the volume of the solution, and *m* (g) is the mass of the material.

The gold recovery kinetics was determined by adding 2 mg of COFs into 500 ml of 16 ppm gold solution of pH 3, and the recovery rate was determined in the dark or under light. The concentration of AuCl_4_^−^ was 20 ppm under different light wavelengths and intensities. The gold recovery was determined by adding 2 mg of COFs into 500 ml of a 12 ppm gold solution at pH 3. After the adsorption reached equilibrium in the dark, the recovery efficiency was subsequently measured under light irradiation. The recovery selectivity was determined in mixture containing Au(III), Al(III), Zn(II), Cd(II), Co(II), Mg(II), Pb(II), Cu(II), and Ni(II), with each concentration of 100 ppm. Then, 5 mg of COFs was added into 20 ml of the above metal ion mixture and the recovery rate was determined under light at room temperature. The reusability was analyzed by adding 10 mg of COFs into a 50-ml 10 ppm gold solution and left to recover for 4 h. The recovered gold was eluted with a mixture containing thiourea (0.25 M) and hydrochloric acid (0.25 M) for 12 h, and the treated COFs were reused for the next gold recovery cycle.

### Gold recovery performance assay from electronic waste

Each waste CPU board was treated with 50 ml of aqua regia for 24 h to elute the metal elements. Then, the leachate was diluted 10 times with pure water and filtered with a 0.22-μm membrane filter. Subsequently, 10 mg of COF was added into 50 ml of the diluted leachate and left to react for 4 h under the light of 1 sun. The metal recovery rates were determined by testing the concentration of metal elements in the solution using ICP-OES. The distribution coefficient (*k*_d_), which is used to determine the recovery selectivity, was calculated as follows.

$$k_{d}=\frac{\left(C_{0}-C_{e}\right)}{C_{e}}\times \frac{V}{m}$$ (3)

where *V* is the volume of the solution (ml), *m* is the amount of material (g), and *C*_0_ and *C*_e_ are the initial concentration and final equilibrium concentration of metal elements, respectively (mg l^−1^).

## Data Availability

All data are available in the manuscript or the Supplementary Materials or from the authors.

## Supplementary Materials

Supplementary 1 Figs. S1 to S27 Tables S1 to S4 References [42–68]
