# Supplementary material for: Electronic Structure Tailored Covalent Organic Frameworks for Synergistic Adsorptive–Photocatalytic Gold Recovery from Complex Electronic Waste
Source: Research (Wash D C). 2025 Nov 24;8:1012. doi: 10.34133/research.1012 (PMC12641164; doi:10.34133/research.1012)
Supplement: Supplementary 1 — Figs. S1 to S27 Tables S1 to S4 References [42–68] [file research.1012.f1.docx]

Supplementary Materials for

**Electronic Structure Tailored Covalent Organic Frameworks for Synergistic Adsorptive–Photocatalytic Gold Recovery from Complex Electronic Waste**

Jun Zhang^1^, Lijuan Feng^1^, Jiacheng Zhang^1^, Jianfei Du^1^, Xuewen Cao^1^, Yan Li^1^, Zhanhu Guo^2^, Yihui Yuan^1*^ & Ning Wang^1*^

^1^School of Marine Sciences, State Key Laboratory of Marine Resource Utilization in South China Sea, Hainan University, Haikou 570228, P. R. China

^2^Department of Mechanical and Civil Engineering, Faculty of Engineering and Environment, Northumbria University, Newcastle Upon Tyne NE1 8ST, UK

*Address correspondence to: Ning Wang; wangn02@foxmail.com and Yihui Yuan; yuanyh@hainanu.edu.cn

**Supplementary Methods**

**Material characterization.** Powder X-ray diffraction (PXRD) tests were performed using a Rigaku Smart Lab diffractometer with Cu Kα radiation (λ = 1.5418 Å). Morphological analysis was conducted using a Hitachi S-4800 scanning electron microscope (SEM) and a Thermo Fisher Verios G4UC field emission transmission electron microscope (FE-TEM). X-ray photoelectron spectroscopy (XPS) was performed using a Thermo escalab 250XI spectrometer. ^13^C nuclear magnetic resonance (NMR) spectra were obtained using an AVANCE NEO 400 NMR spectrometer. Infrared absorption spectra were collected using a Fourier-transform infrared spectrometer (FT-IR) LR-64912C. Metal ion quantification was determined by inductively coupled plasma–optical emission spectroscopy (ICP-OES) using an Agilent 5800 VDV spectrometer. UV-visible diffuse reflectance spectra (UV/vis DRS) were measured using a UV-3600 UV-Vis-NIR spectrophotometer. Nitrogen adsorption isotherms were measured using a 3flex adsorption system at 77 K. The Brunauer-Emmett-Teller (BET) surface area was calculated based on the nitrogen adsorption isotherms. The contact angles were measured using a JC2000D instrument. Thermogravimetric analysis (TGA) was conducted using a TL9000 analyzer. Electron paramagnetic resonance (EPR) spectra of oxygen vacancies were obtained using a BRUKER ESR5000 spectrometer. Room temperature steady-state photoluminescence (PL) measurements were performed using a FLS1000/FS5 system with a 585 nm laser excitation source. The structural models were generated using Materials Studio 2017/R2. Geometry optimization was performed using the Forcite module. The Reflex module was used for full-profile pattern fitting (Pawley) of the experimental powder diffraction pattern.

**Powder X-Ray diffraction.**

Powder X-ray diffraction was tested on Rigaku Smart Lab with a Cu Kα source (λ =1.5418 Å) at 40 kV and 75 mA. The optics slit was set to 10 mm. The samples were ground into powder and evenly applied to the sample groove. Samples are tested from 2 to 80 degrees with a scan speed of 5 degrees per minute.

**Fourier-transform infrared spectrometer.**

Infrared absorption spectra were collected using a LR-64912C. The sample was mixed and ground with KBr in a ratio of 10:1, then pressed into thin sheet with hydraulic press. The spectra were recorded between 4000 and 400 cm^−1^ at a resolution of 4 cm^−1^.

**X-ray photoelectron spectroscopy.**

X-ray photoelectron spectroscopy measurements were tested by Thermo escalab 250XI. Experiments were performed with a beam size of 650 µm using a monochromatic Al Kα X-ray source of 150 W power. The data were analyzed by Avantage.

**Nitrogen adsorption and desorption.**

All nitrogen adsorption and desorption measurements were performed on a 3Flex 5.02 instrument. The samples were first dried at 80 °C in a vacuum oven overnight. About 0.1 g of samples were activated at 120 °C by the pretreatment system for 8 h. After activation, the samples were cooled to room temperature, back-filled with nitrogen. Nitrogen adsorption isotherms were tested at 77 K with nitrogen 99.999% purity gas using the BET (Brunauer-Emmet-Teller) method to calculate the surface area.

**Electron microscopy.**

Scanning electron Microscopy (SEM) characterizations were performed on Hitachi S-4800. The samples were evenly coated on the silicon plate. The acceleration voltage of electronic source was adjusted to 15 kV.

Scanning transmission electron microscopy (FE-TEM) images were collected by Thermo Fisher Verios G4UC and corresponding FE-TEM-EDS spectroscopy was performed.

**Photoluminescence.**

The emission spectra were collected using an FLS1000 transient steady-state fluorescence spectrometer. A 20 mg sample was ground and then hydraulically pressed into a thin sheet. The spectra were recorded between 605 and 851 cm^−1^. Fluorescence lifetime data were collected under excitation at 585 cm^−1^.

**Electrochemical property measurements**

The photocurrent response was measured using an electrochemical workstation (Shanghai CHI-700D) under xenon lamp irradiation with a 400 nm cutoff filter. The photo-catalyst-coated ITO glass (1 cm × 1 cm), Pt plate, Ag/AgCl, and 0.5 M Na_2_SO_4_ aqueous solution were used as the working electrode, counter electrode, reference electrode, and electrolyte, respectively. The preparation of the working electrode is as follows. COF powder (20 mg) was dissolved in 1 mL of anhydrous ethanol and then ground until a slurry was formed. The slurry was then coated onto the ITO glass and dried. Afterward, 10 μL of Nafion solution was dropped onto the ITO glass. The Mott-Schottky experiment was recorded on the electrochemical workstation with frequencies of 1000 Hz, 2000 Hz, and 3000 Hz. Electrochemical impedance measurements were performed under dark conditions with a frequency range of 0.1 Hz to 100 kHz and an amplitude of 5 mV. Ag/AgCl were converted to normal hydrogen electrode (NHE) potentials using the following equation, E_NHE_ = E_Ag/AgCl_ + E^θ^_Ag/AgCl_ (E^θ^_Ag/AgCl_ = 0.199 V), where E_Ag/AgCl_ is the tested potential.

**Density functional theory (DFT) calculations**

Quantum chemical studies are performed using density functional theory (DFT) and time-dependent density functional theory (TD-DFT) implemented in GAUSSIAN 16 package1. Geometry optimization and frequency analysis were carried out using the B3LYP hybrid functional, incorporating GD3BJ dispersion correction, and based on the 6-311g(d,p) basis set [42]. After molecular optimization, no imaginary frequencies were observed. Frontier molecular orbitals and electrostatic potential (ESP) were analyzed using the Multiwfn 3.83 [43] and VMD v1.9.34 [44] molecular visualization software. Carbon (C), hydrogen (H), oxygen (O), nitrogen (N), sulfur (S), chlorine (Cl) atoms were computed using the 6-311G(d,p) basis set, while gold (Au), copper (Cu), and nickel (Ni) were treated with the lanl2dz basis set [45, 46]. Vibrational frequency analysis was computed to ensure the minimum have no imaginary frequency, and the value of free energy can be obtained. The formula for calculating the binding energy (AB) of the molecule is as follows: E_b_ = E_(AB)_ - E_(A)_ - E_(B)_. The TD-DFT calculation parameters were set as TD (nstates = 10) and IOp (9/40 = 4). The electron–hole analysis was carried out using the Multiwfn 3.8 program, and molecular visualization was performed with VMD 1.9.3 software.

**Supplementary Figures**


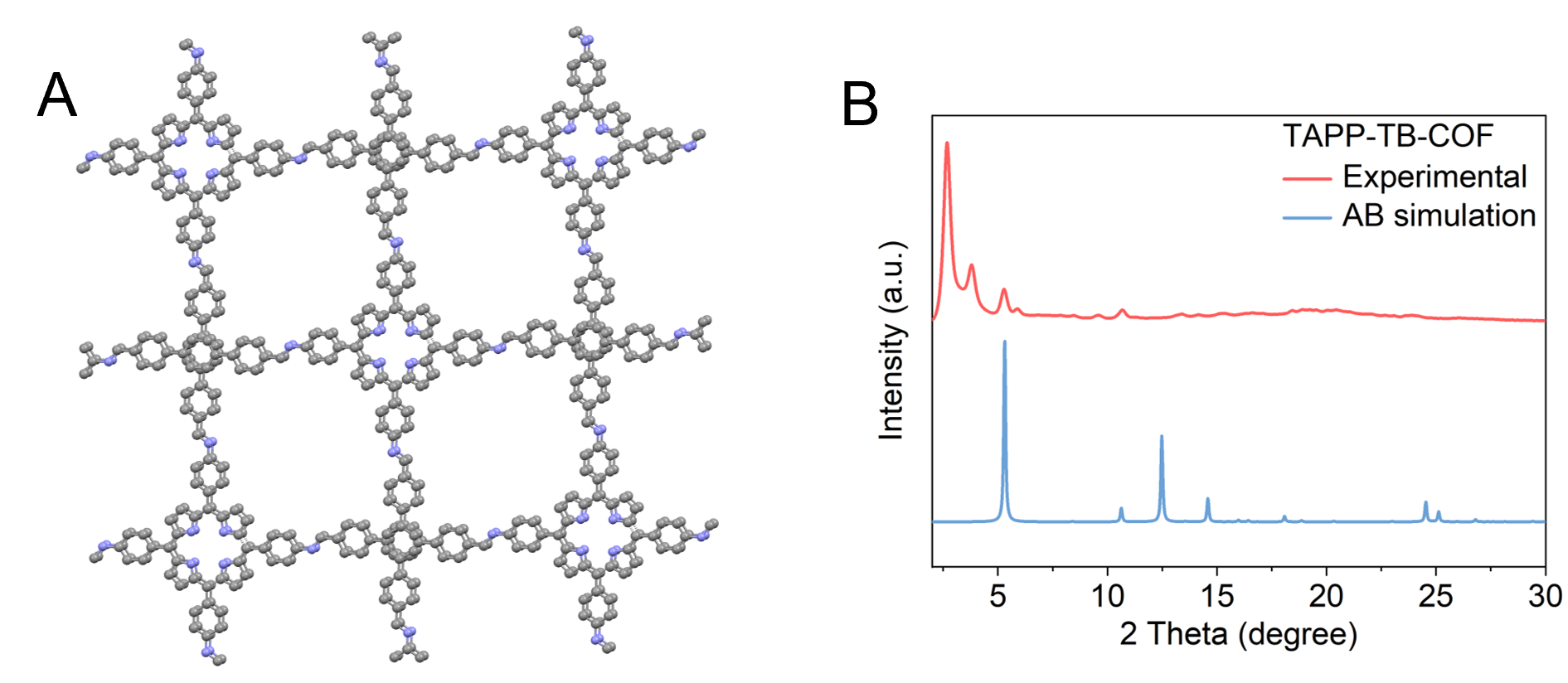


**Fig. S1.** The simulated (A) AB stacking model of TAPP-TB-COF and the experimental and simulated (B) PXRD patterns.


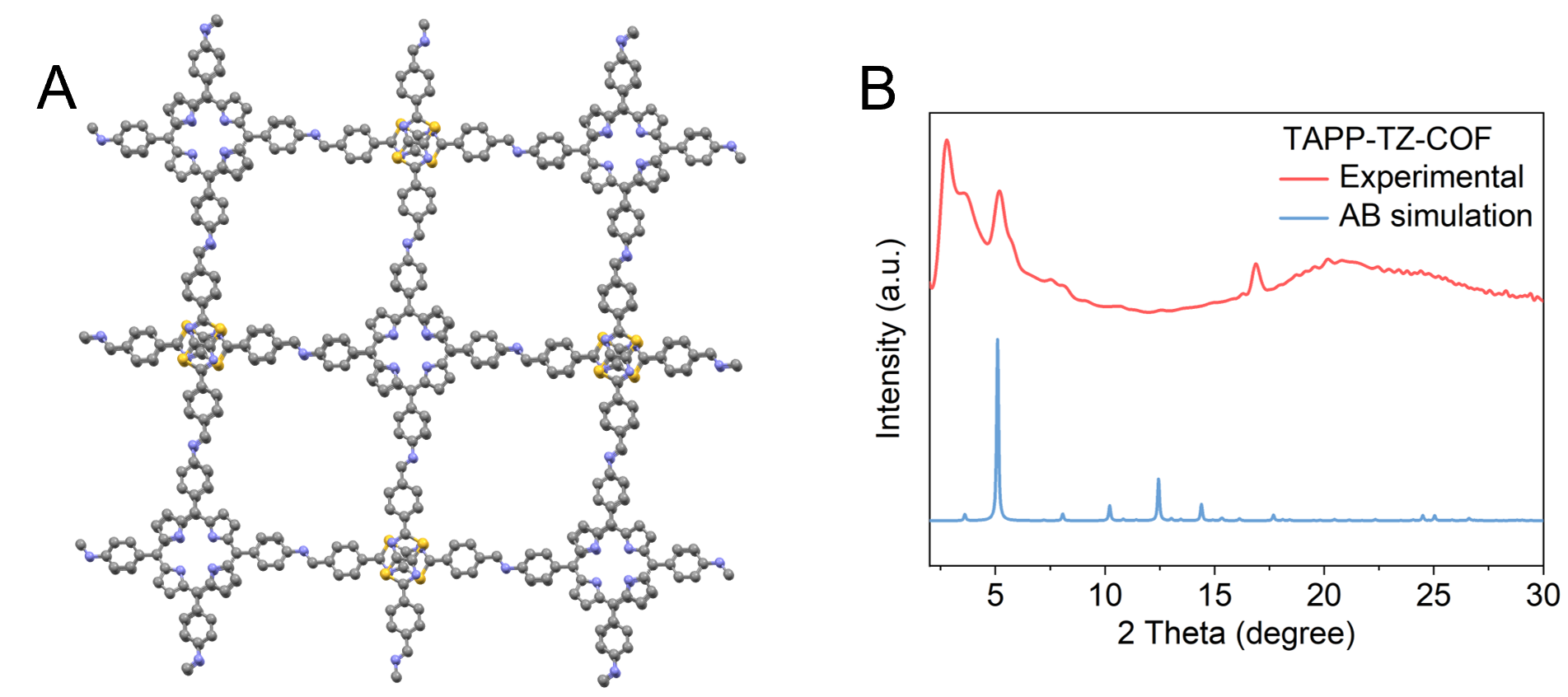


**Fig. S2.** The simulated (A) AB stacking model of TAPP-TZ-COF and the experimental and simulated (B) PXRD patterns.


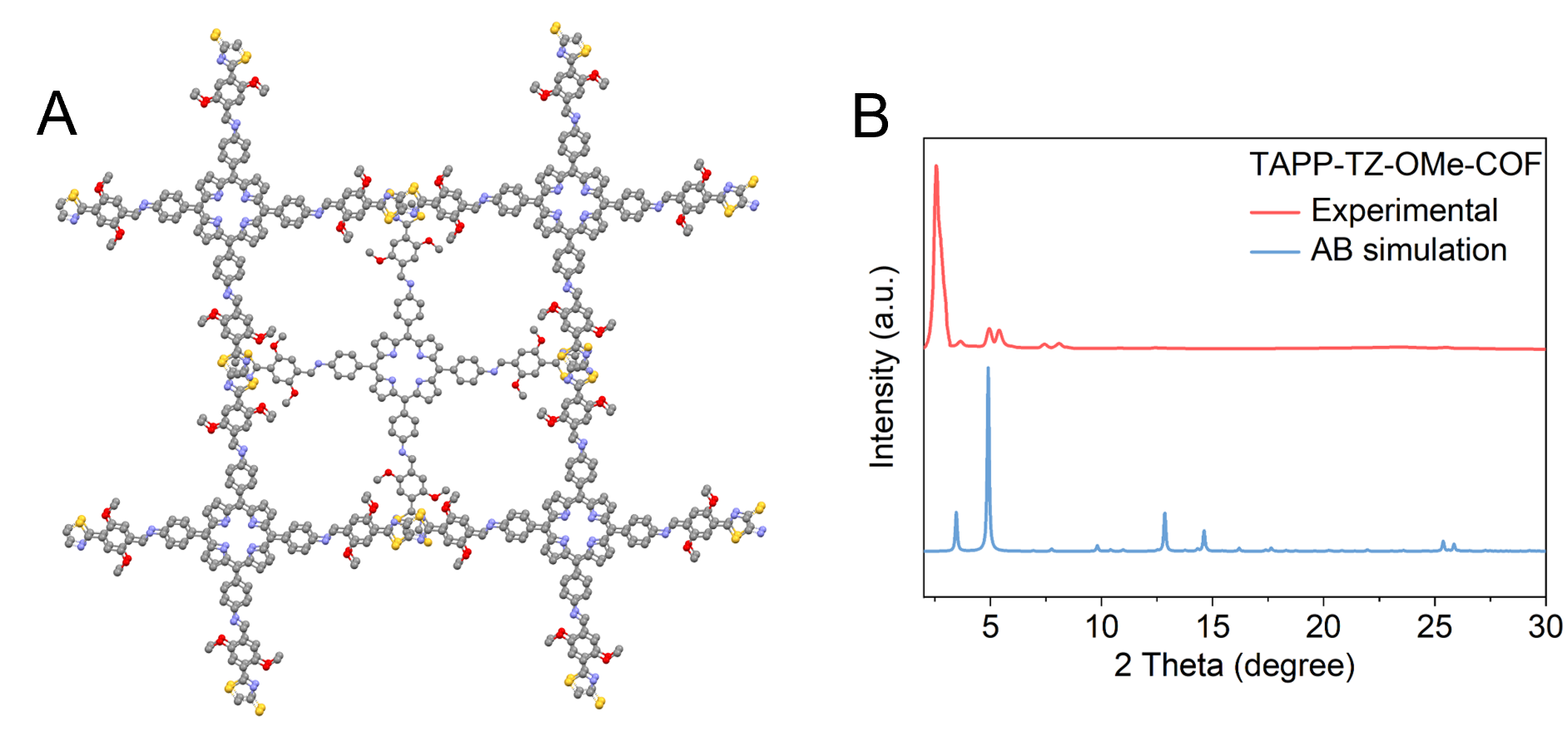


**Fig. S3.** The simulated (A) AB stacking model of TAPP-TZ-OMe-COF and the experimental and simulated (B) PXRD patterns.


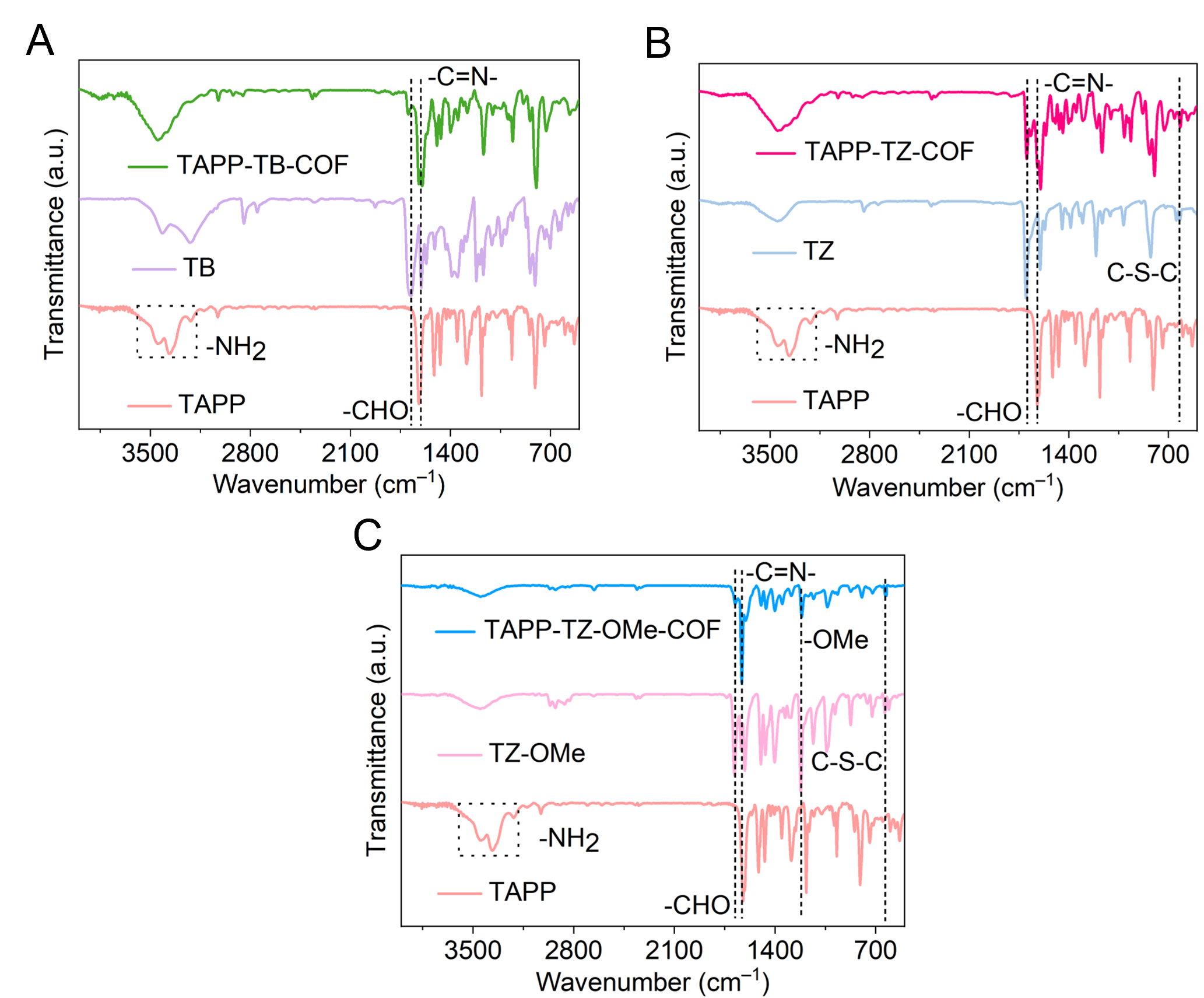


**Fig. S4.** (A) FT-IR spectra of the TAPP, TB, and TAPP-TB-COF. (B) FT-IR spectra of TAPP, TZ, and TAPP-TZ-COF. (C) FT-IR spectra of TAPP, TZ-OMe, and TAPP-TZ-OMe-COF.


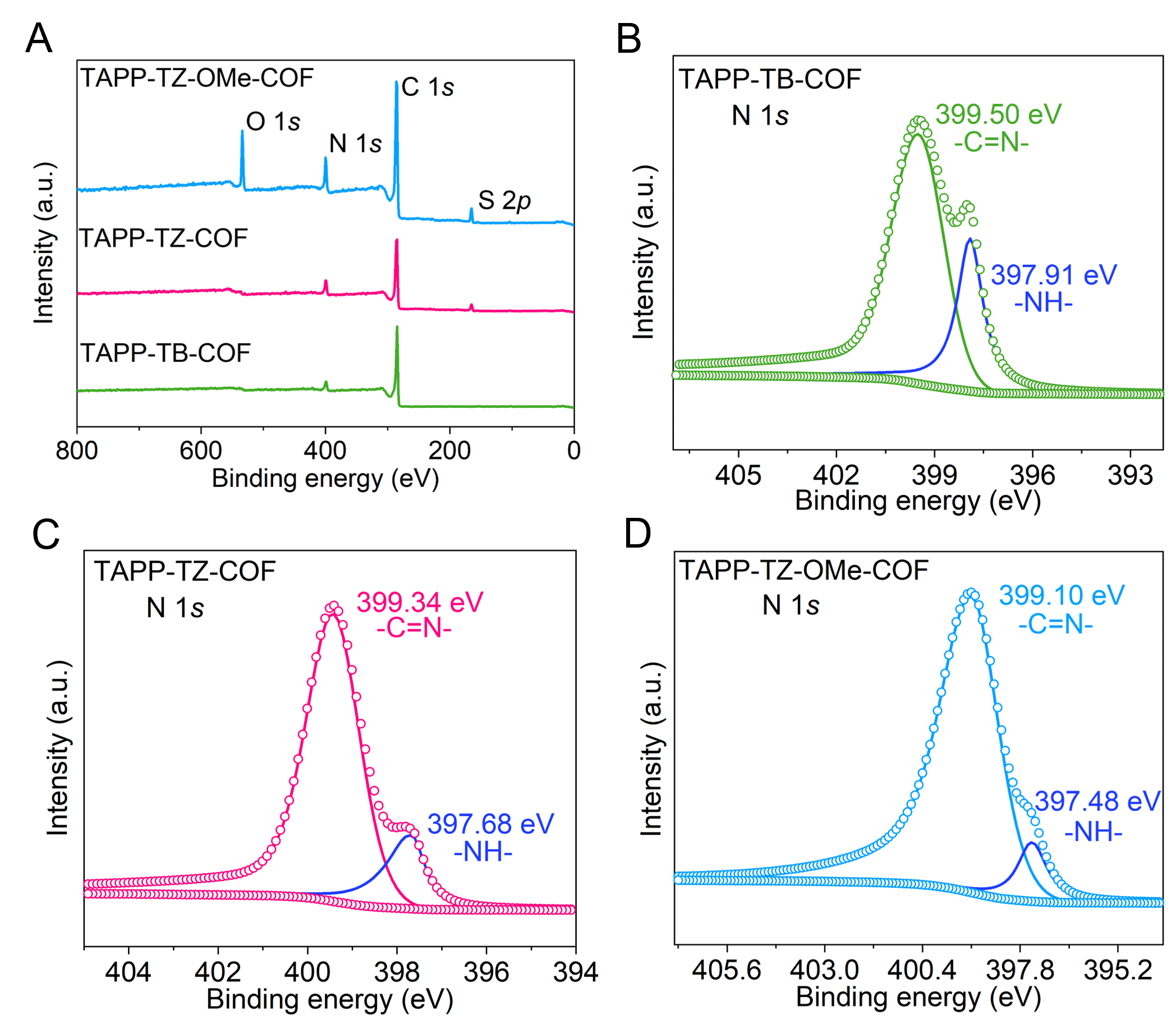


**Fig. S5.** (A) XPS spectra of COFs. XPS spectra of N 1*s* for (B) TAPP-TB-COF, (C) TAPP-TZ-COF, (D) TAPP-TZ-OMe-COF.


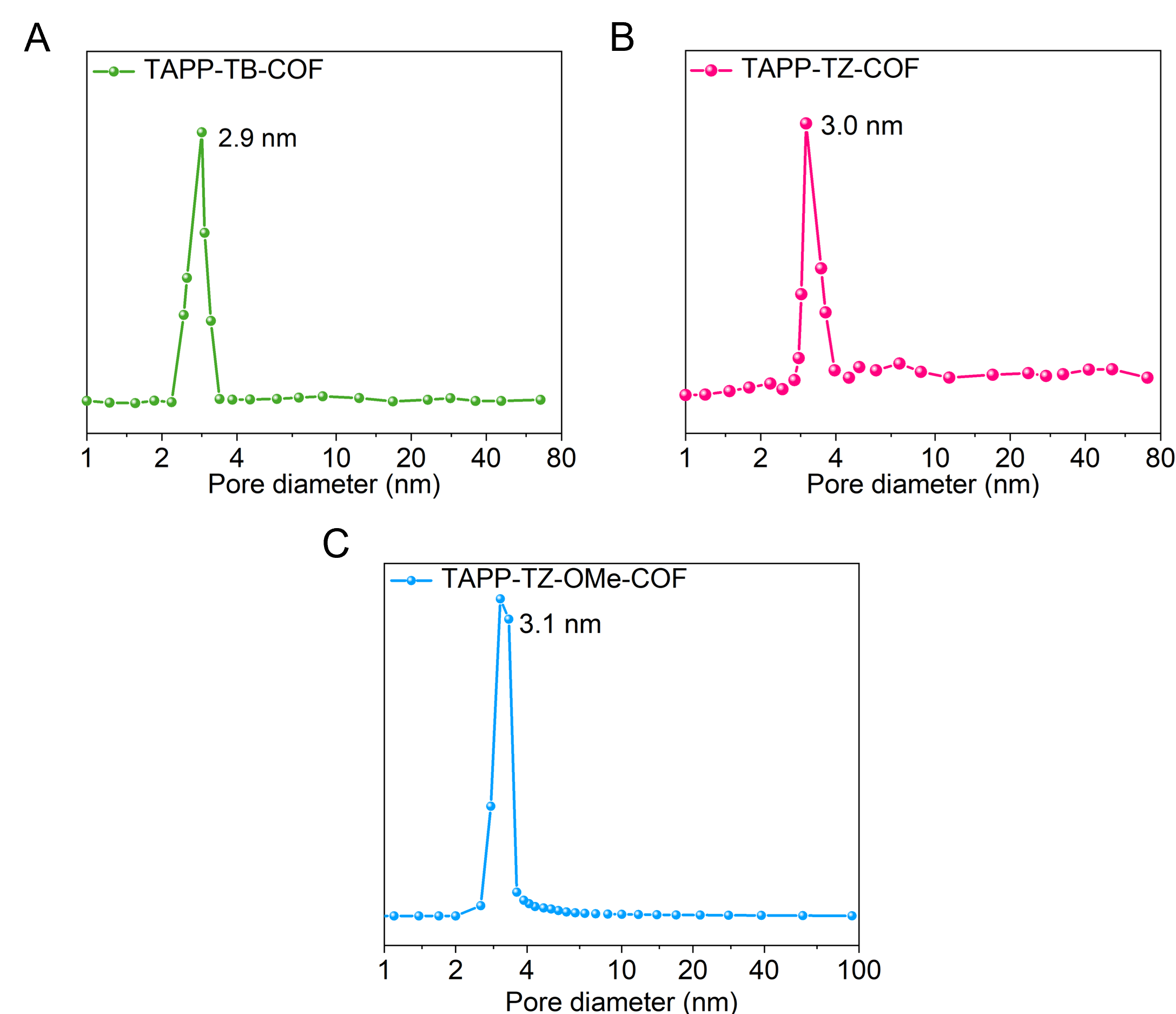


**Fig. S6.** Pore size distribution of (A) TAPP-TB-COF, (B) TAPP-TZ-COF, and (C) TAPP-TZ-OMe-COF determined by N_2_ adsorption-desorption isotherms analysis.


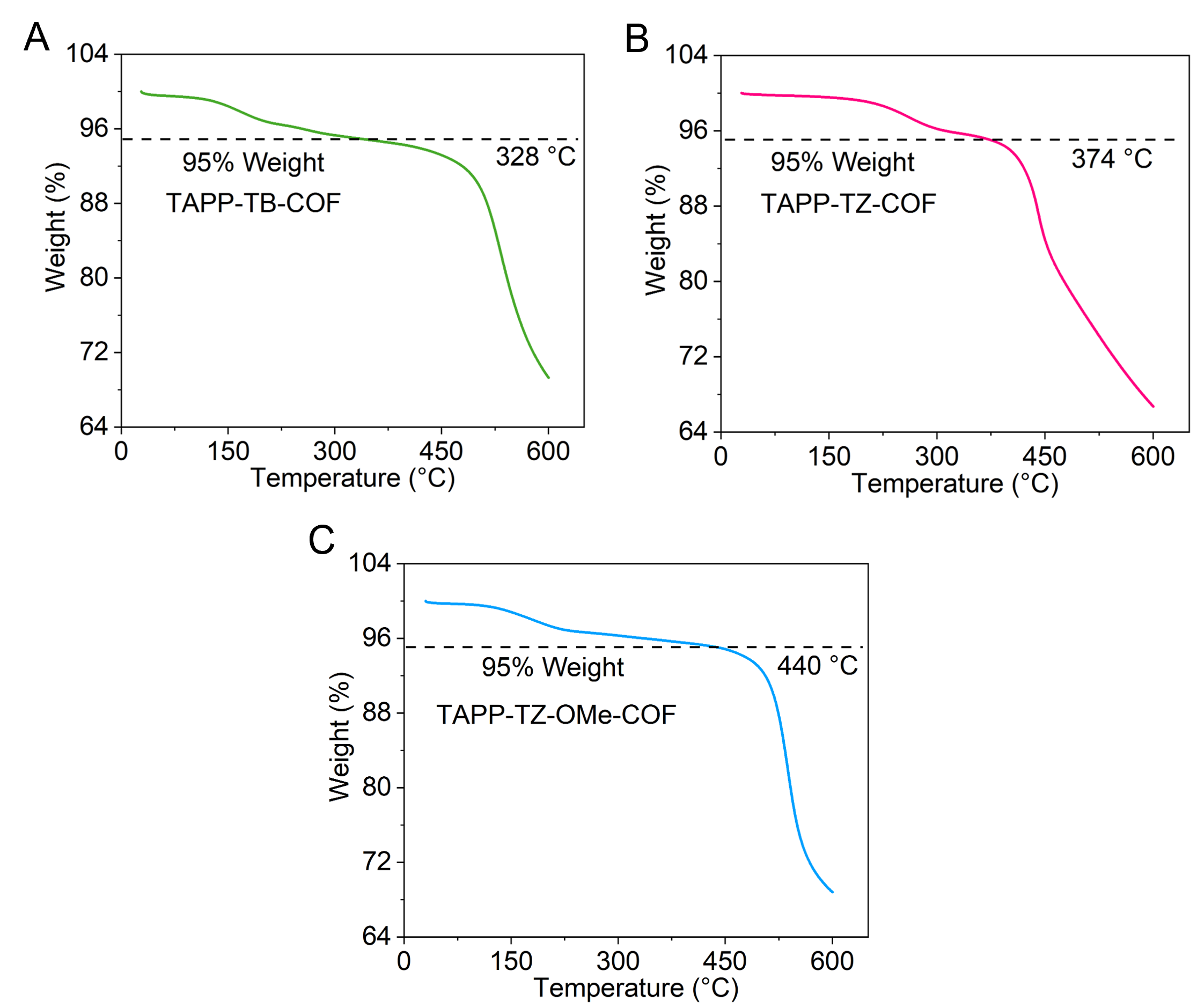


**Fig. S7.** TGA data of (A) TAPP-TB-COF, (B) TAPP-TZ-COF and (C) TAPP-TZ-OMe-COF under N_2_ atmosphere.


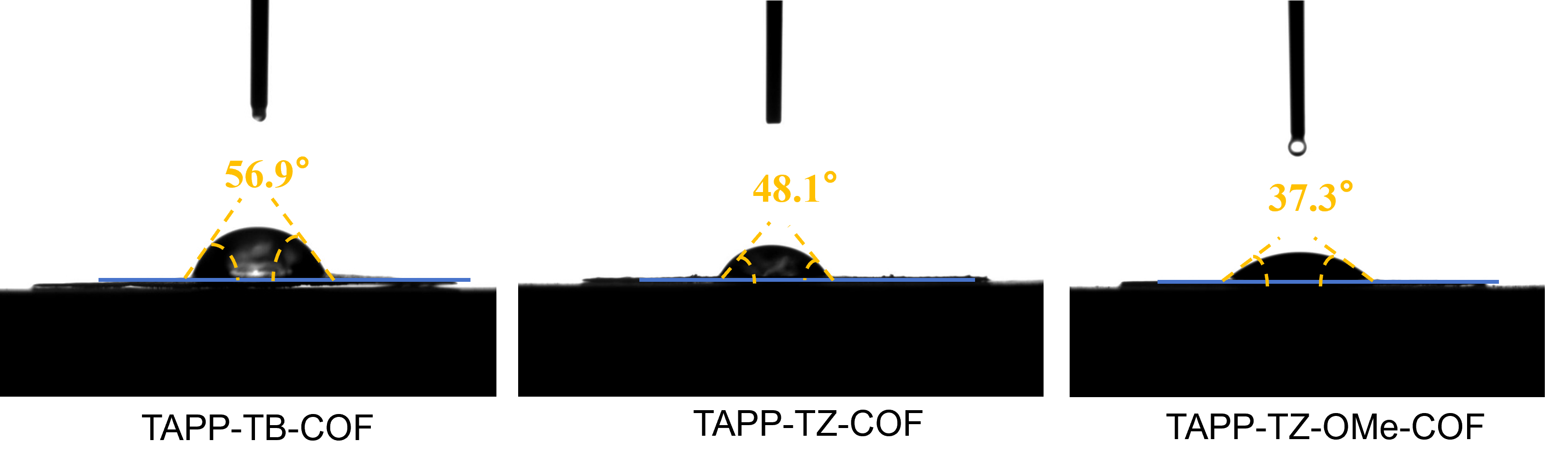


**Fig. S8.** Water contact angle of TAPP-TB-COF, TAPP-TZ-COF, and TAPP-TZ-OMe-COF.

**Fig. S9.** Influence of pH on the gold recovery performance of TAPP-TB-COF, TAPP-TZ-COF, and TAPP-TZ-OMe-COF.


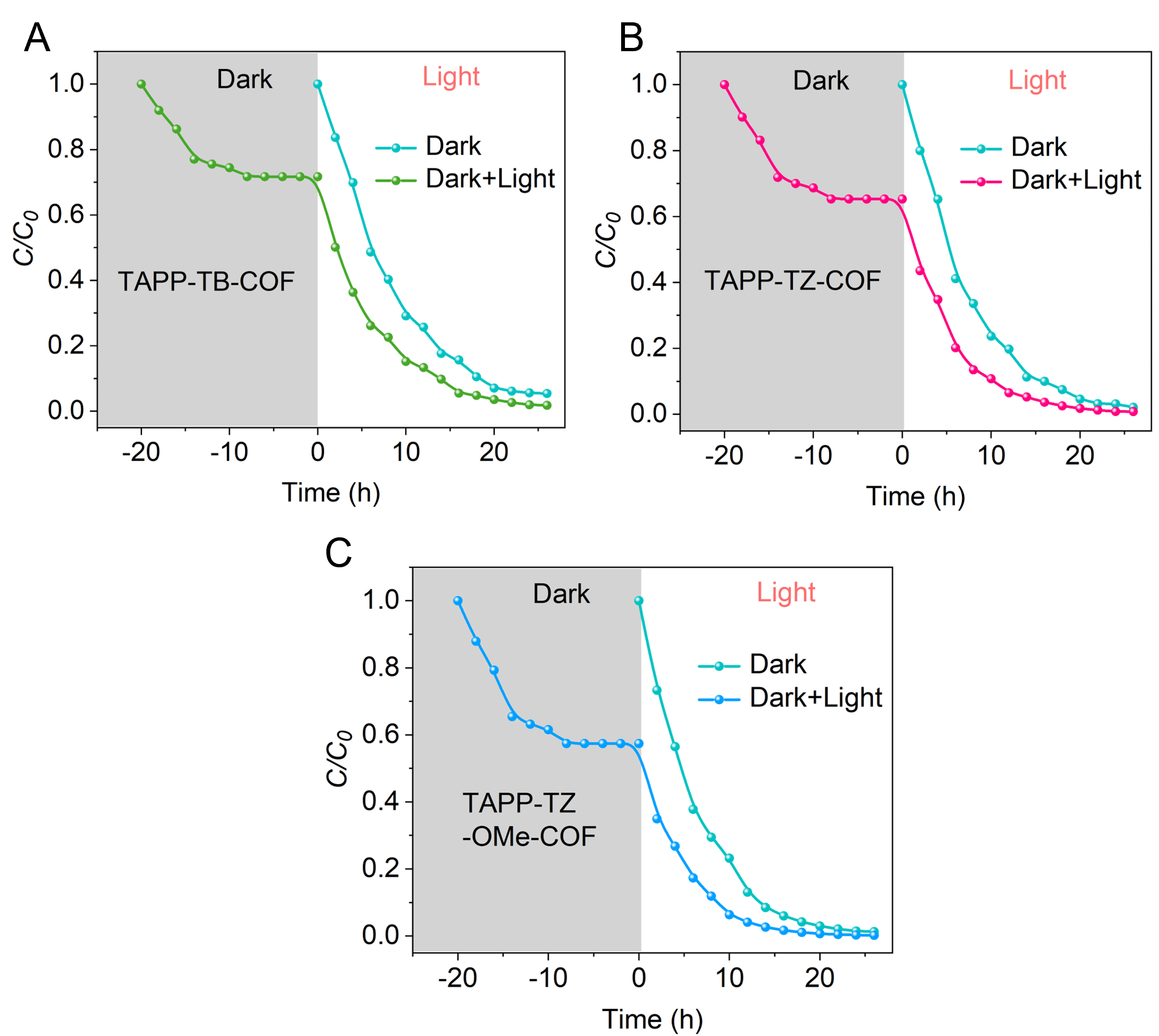


**Fig. S10.** (A) Gold recovery kinetics of TAPP-TB-COF under dark and light conditions. (B) Gold recovery kinetics of TAPP-TZ-COF under dark and light conditions. (C) Gold recovery kinetics of TAPP-TZ-OMe-COF under dark and light conditions.


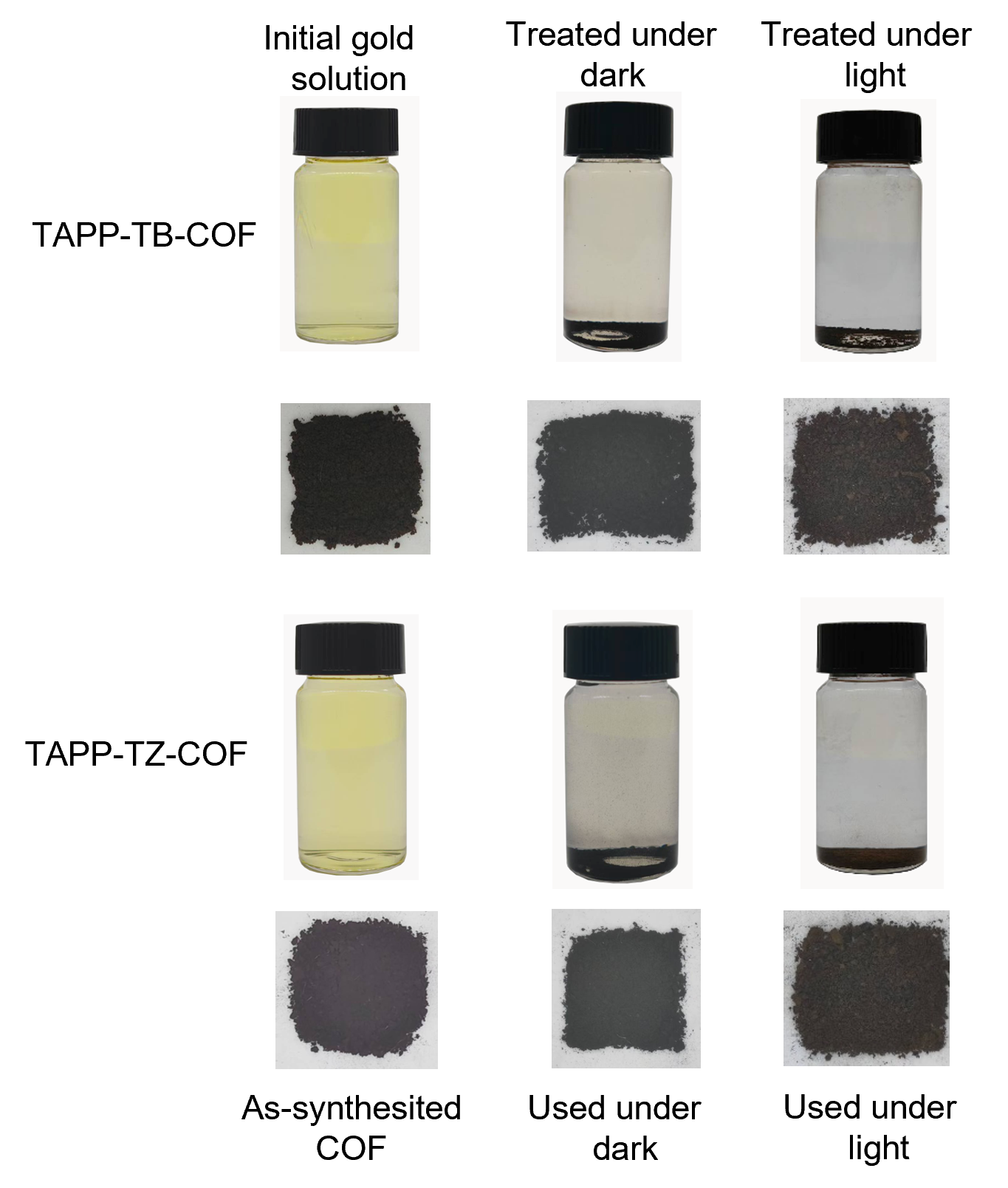


**Fig. S11.** Morphologies of TAPP-TB-COF and TAPP-TZ-COF before and after being used for gold recovery.


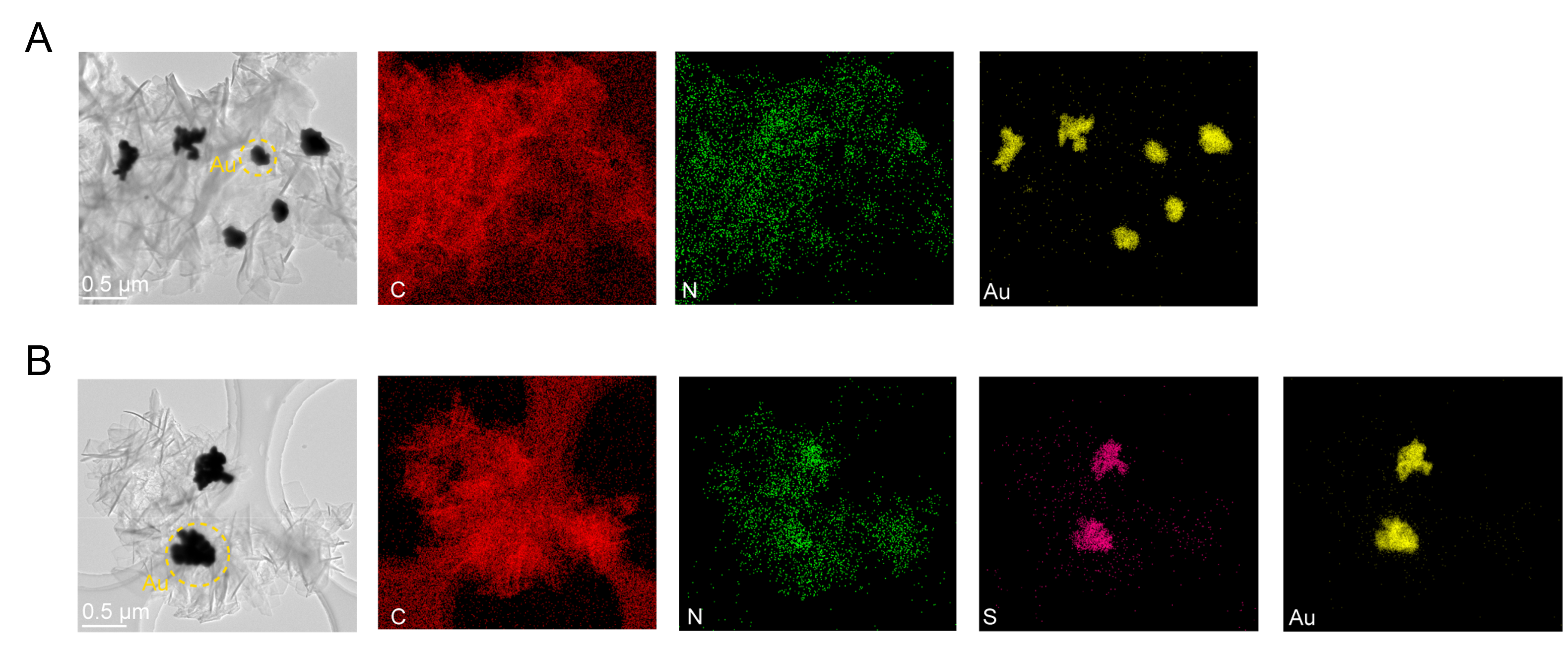


**Fig. S12.** (A) FE-TEM image and corresponding EDS elemental mapping of TAPP-TB-COF after being used for gold recovery under light irradiation. (B) FE-TEM image and corresponding EDS elemental mapping of TAPP-TZ-COF after being used for gold recovery under light irradiation.


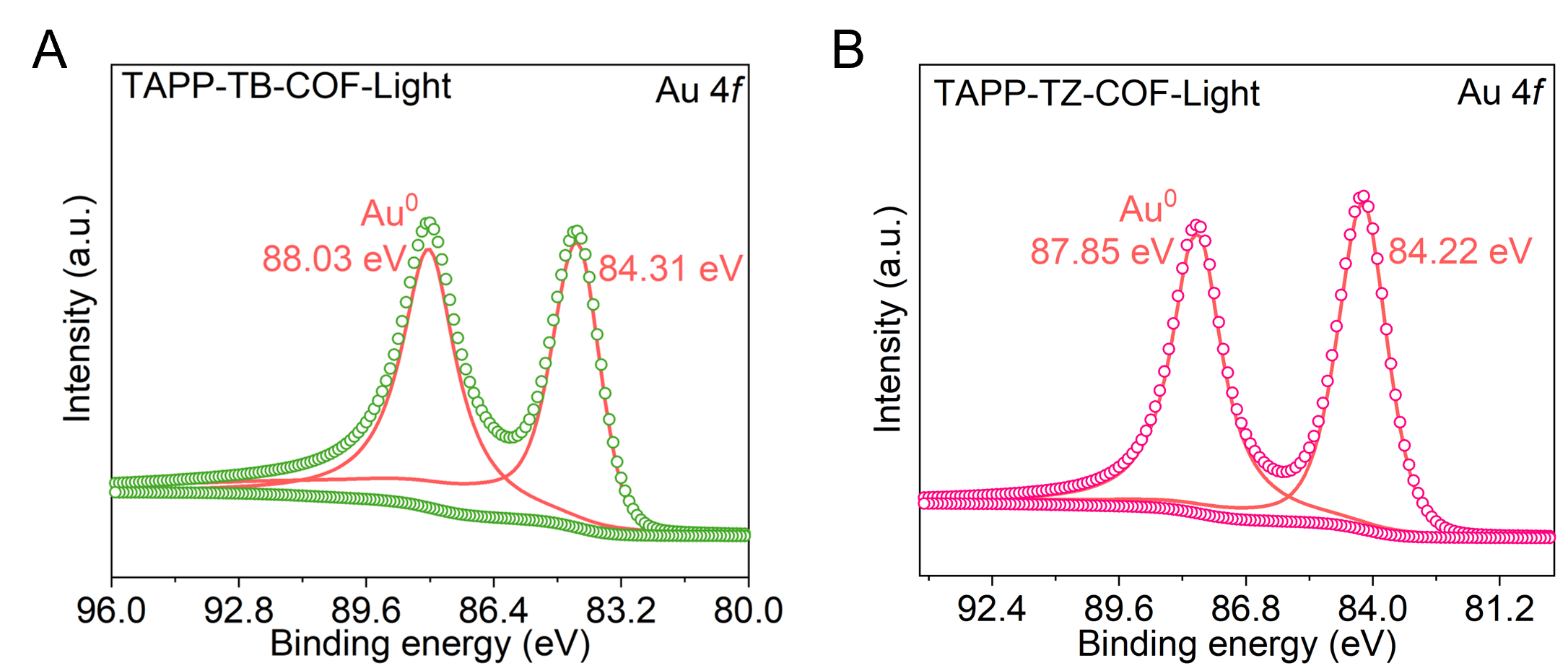


**Fig. S13.** (A) XPS spectrum of Au 4*f* after being recovered by TAPP-TB-COF under light irradiation. (B) XPS spectrum of Au 4*f* after being recovered by TAPP-TZ-COF under light irradiation.


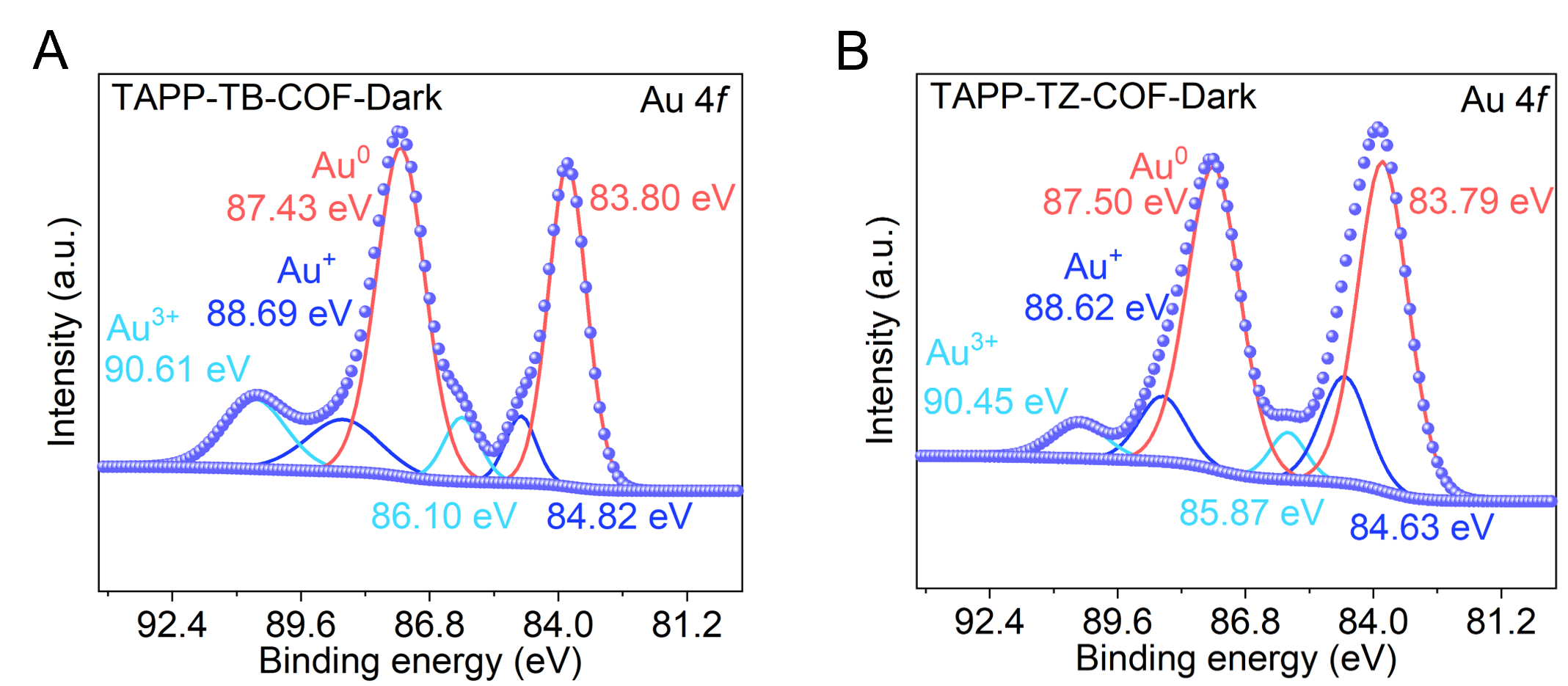


**Fig. S14.** (A) XPS spectrum of Au 4*f* after being recovered by TAPP-TB-COF under dark condition. (B) XPS spectrum of Au 4*f* after being recovered by TAPP-TZ-COF under dark condition.

**
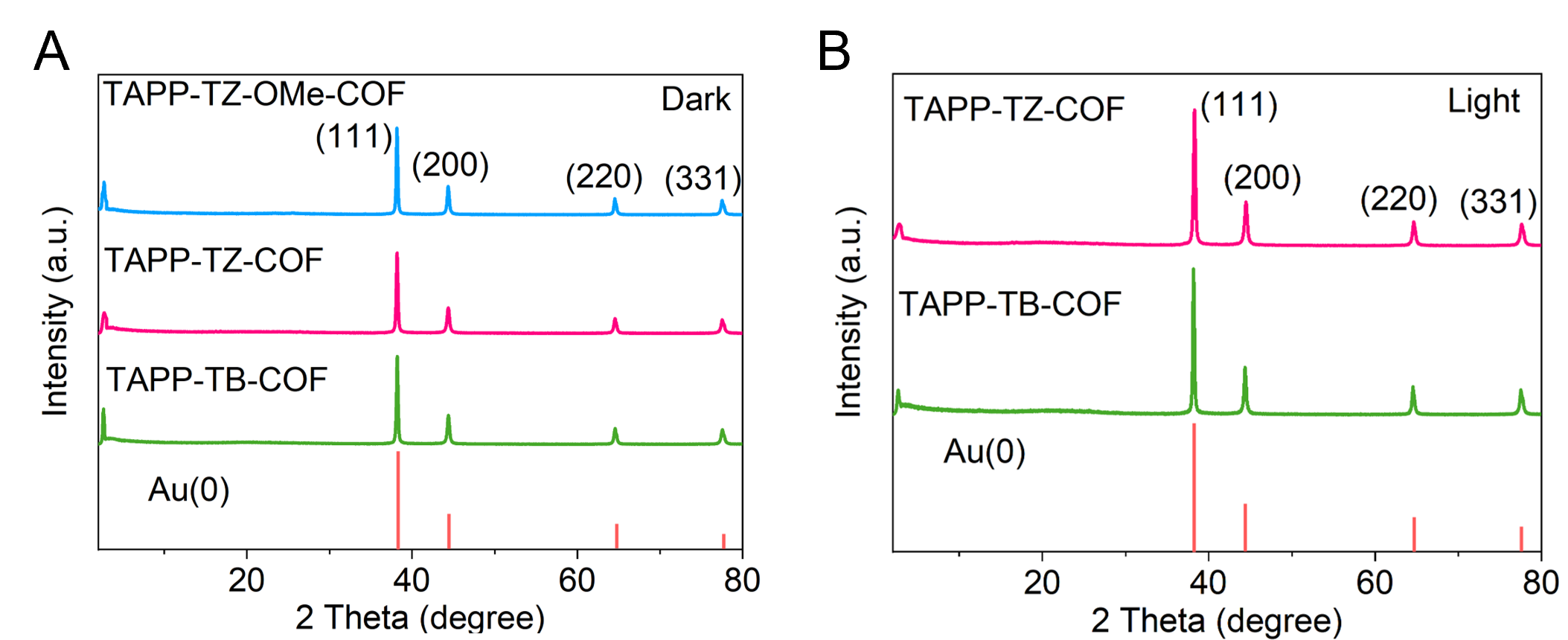
**

**Fig. S15.** (A) PXRD spectra of COFs after being used for gold recovery under dark conditions. (B) PXRD spectra of TAPP-TB-COF and TAPP-TZ-COF after being used for gold recovery under light irradiation.

**Fig. S16.** Gold elution kinetics from used TAPP-TZ-OMe-COF.

**Fig. 17.** Comparison of the binding energies of specific coordination sites in TAPP-TZ-OMe-COF toward Au(III), Cu(II), and Ni(II).

**Fig. S18.** XPS spectra of N 1*s* from TAPP-TB-COF before and after being used for gold recovery under dark condition.


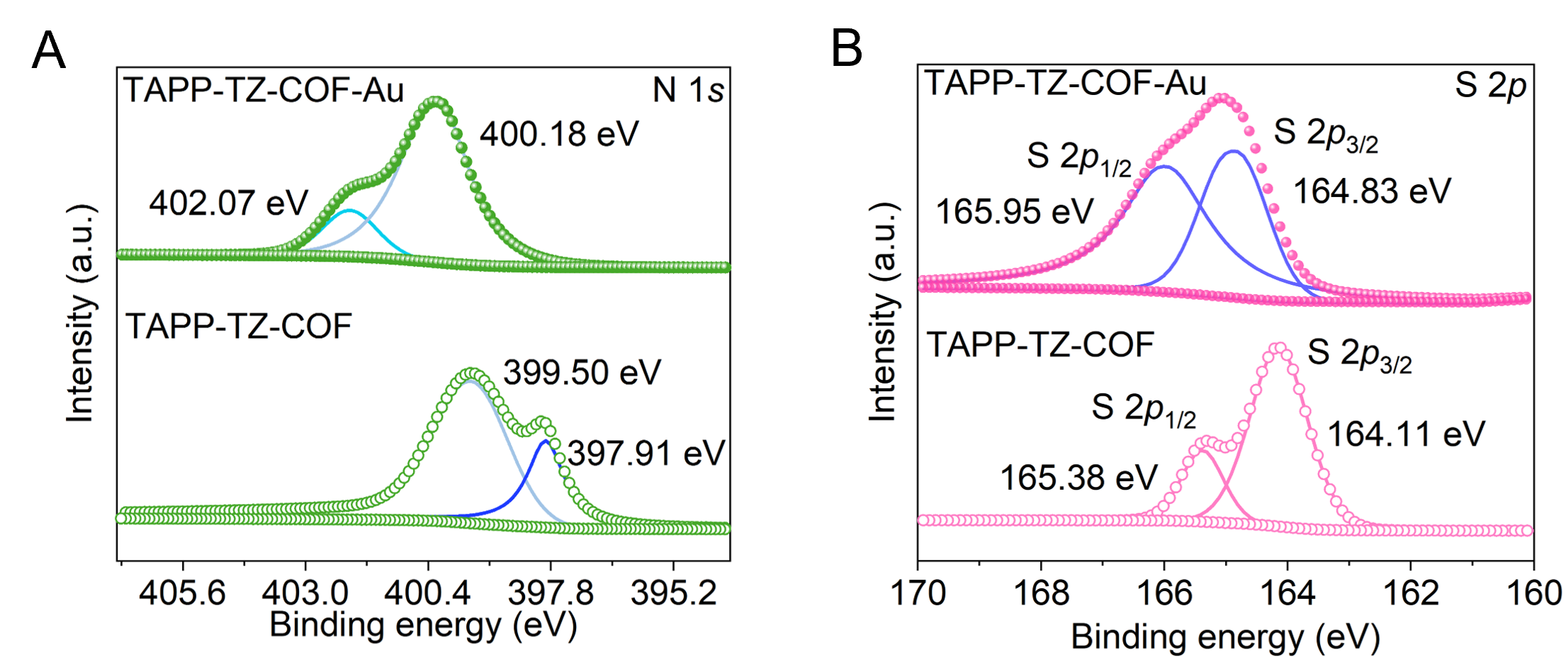


**Fig. S19.** (A) XPS spectra of N 1*s* from TAPP-TZ-COF before and after being used for gold recovery under dark condition. (B) XPS spectra of S 2*p* from TAPP-TZ-COF before and after being used for gold recovery under dark condition.

**Fig. S20.** EPR spectra of TAPP-TZ-OMe-COF and gold-loaded TAPP-TZ-OMe-COF under dark condition.

**Fig. S21.** UV/vis diffuse reflectance spectra of TAPP-TB-COF, TAPP-TZ-COF, and TAPP-TZ-OMe-COF.


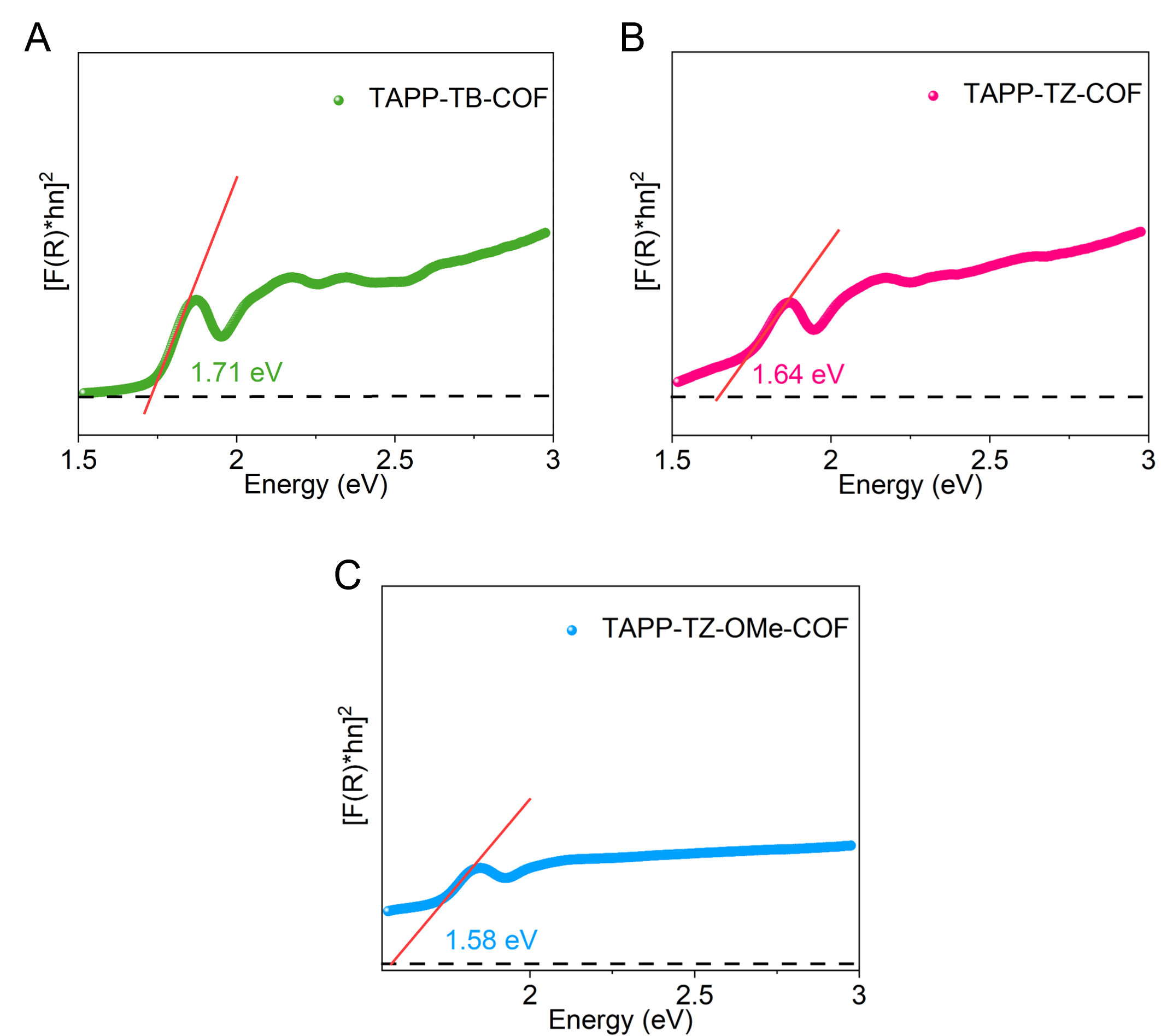


**Fig. S22.** Tauc’ plots of (A) TAPP-TB-COF, (B) TAPP-TZ-COF, and (C) TAPP-TZ-OMe-COF.


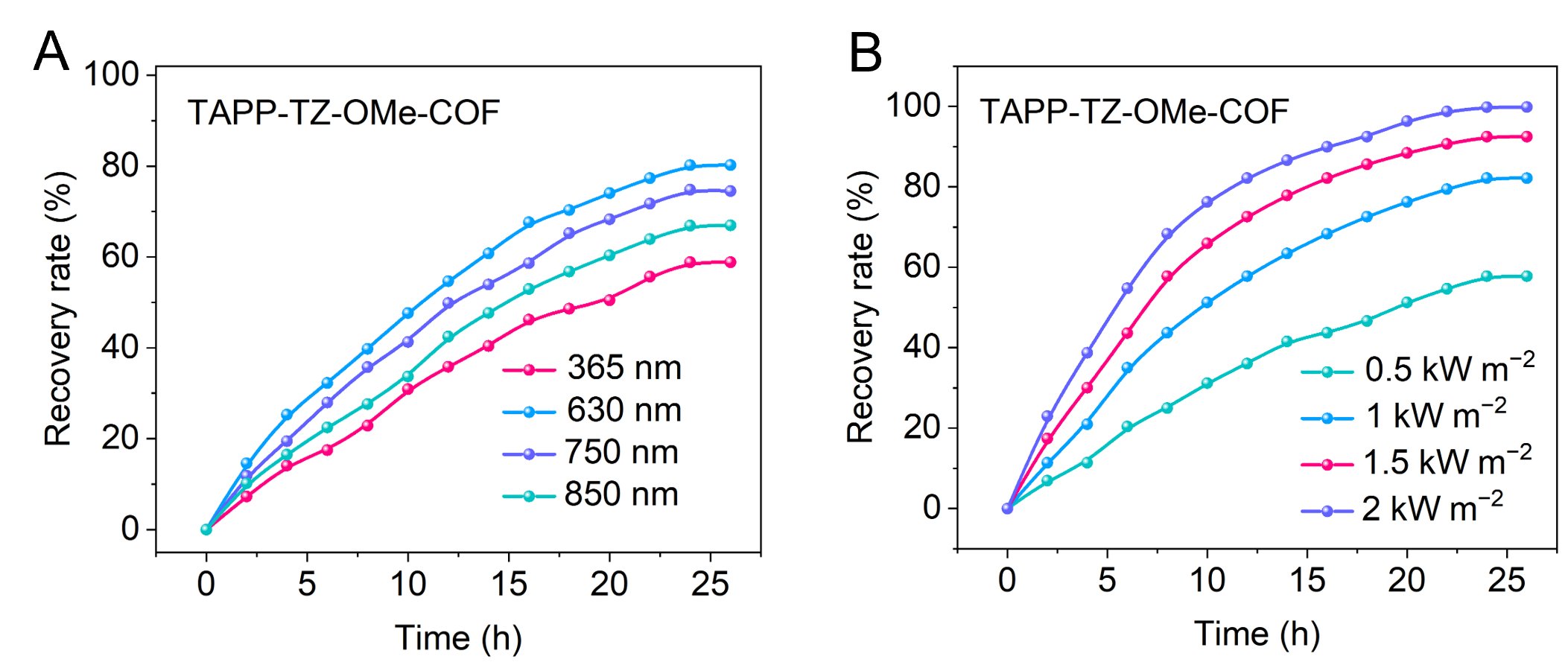


**Fig. S23.** (A) Gold recovery performance of TAPP-TZ-OMe-COF under different light wavelengths. (B) Gold recovery performance of TAPP-TZ-OMe-COF under different light intensities.

**Fig. S24.** Photocurrent response of COFs under visible light irradiation.

**Fig. S25.** EIS spectra of COFs.

**Fig. S26.** PL spectra of COFs excited by light of 585 nm.

**
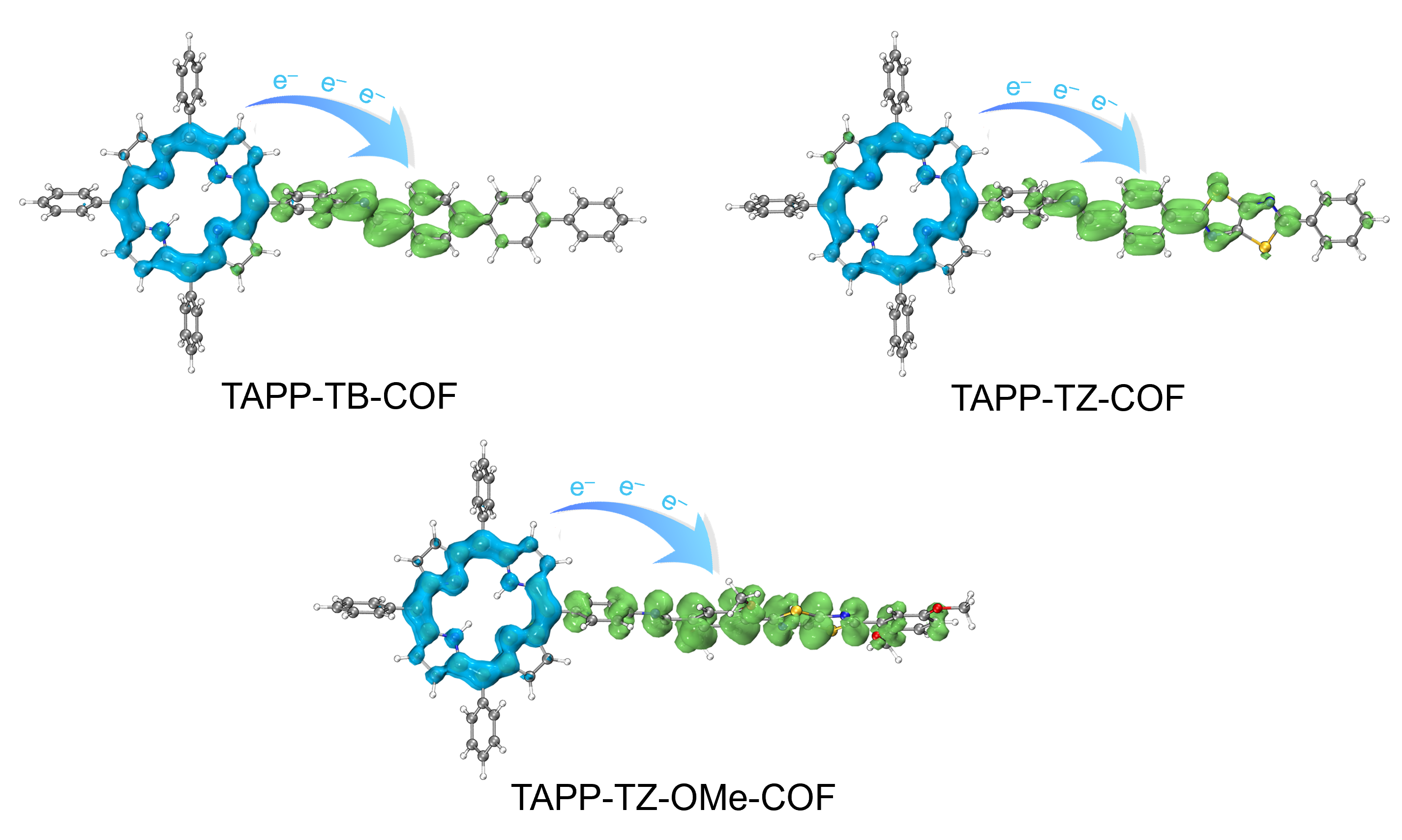
**

**Fig. S27.** Time-dependent density functional theory (TD-DFT) diagram of electron transfer within the structural units of the COFs.

**Table S1.** AA stacking model of fractional atomic coordinates for TAPP-TB-COF.

| TAPP-TB-COF  Space group: *P*1–triclinic  a=33.4241 Å, b=33.2071 Å, c=3.6257 Å  α=90.0000°, β=90.0000°, γ=90.0000° | | | |
| --- | --- | --- | --- |
|  |  |  |  |
| Atom | x | y | z |
| C1 | 1.46213 | -1.29672 | -0.5 |
| C2 | 1.46389 | -1.33757 | -0.5 |
| C3 | 1.49734 | -1.35749 | -0.5 |
| C4 | 1.52913 | -1.33523 | -0.5 |
| C5 | 1.52765 | -1.29437 | -0.5 |
| C6 | 1.49398 | -1.27486 | -0.5 |
| C7 | 1.49901 | -1.4014 | -0.5 |
| C8 | 1.46488 | -1.4232 | -0.5 |
| C9 | 1.43058 | -1.40714 | -0.5 |
| C10 | 1.4034 | -1.43672 | -0.5 |
| C11 | 1.42232 | -1.46953 | -0.5 |
| N12 | 1.46017 | -1.46158 | -0.5 |
| C13 | 1.4035 | -1.50536 | -0.5 |
| C14 | 1.42526 | -1.53931 | -0.5 |
| C15 | 1.35961 | -1.50735 | -0.5 |
| C16 | 1.33707 | -1.47567 | -0.5 |
| C17 | 1.29624 | -1.47734 | -0.5 |
| C18 | 1.277 | -1.51108 | -0.5 |
| C19 | 1.29913 | -1.54286 | -0.5 |
| C20 | 1.33998 | -1.54091 | -0.5 |
| C21 | 1.40829 | -1.57586 | -0.5 |
| C22 | 1.4367 | -1.60197 | -0.5 |
| C23 | 1.47166 | -1.58196 | -0.5 |
| N24 | 1.46369 | -1.54403 | -0.5 |
| C25 | 1.5073 | -1.60076 | -0.5 |
| C26 | 1.54142 | -1.57897 | -0.5 |
| C27 | 1.57573 | -1.59503 | -0.5 |
| C28 | 1.60291 | -1.56545 | -0.5 |
| C29 | 1.58399 | -1.53264 | -0.5 |
| N30 | 1.54614 | -1.54059 | -0.5 |
| C31 | 1.60281 | -1.4968 | -0.5 |
| C32 | 1.58105 | -1.46285 | -0.5 |
| C33 | 1.59801 | -1.4263 | -0.5 |
| C34 | 1.56961 | -1.40019 | -0.5 |
| C35 | 1.53465 | -1.4202 | -0.5 |
| N36 | 1.54262 | -1.45813 | -0.5 |
| C37 | 1.6467 | -1.49481 | -0.5 |
| C38 | 1.50897 | -1.64467 | -0.5 |
| C39 | 1.47717 | -1.66693 | -0.5 |
| C40 | 1.47866 | -1.7078 | -0.5 |
| C41 | 1.51233 | -1.7273 | -0.5 |
| C42 | 1.54418 | -1.70545 | -0.5 |
| C43 | 1.54242 | -1.6646 | -0.5 |
| C44 | 1.66924 | -1.5265 | -0.5 |
| C45 | 1.71007 | -1.52482 | -0.5 |
| C46 | 1.7293 | -1.49108 | -0.5 |
| C47 | 1.70718 | -1.45931 | -0.5 |
| C48 | 1.66633 | -1.46126 | -0.5 |
| N49 | 1.49128 | -1.23304 | -0.5 |
| N50 | 1.77112 | -1.48828 | -0.5 |
| N51 | 1.51503 | -1.76913 | -0.5 |
| C52 | 1.79382 | -1.51762 | -0.5 |
| C53 | 1.83652 | -1.51356 | -0.5 |
| C54 | 1.85965 | -1.54688 | -0.5 |
| C55 | 1.90044 | -1.54383 | -0.5 |
| C56 | 1.91869 | -1.50735 | -0.5 |
| C57 | 1.89524 | -1.47398 | -0.5 |
| C58 | 1.85445 | -1.47704 | -0.5 |
| C59 | 1.96192 | -1.50414 | -0.5 |
| C60 | 1.98538 | -1.53787 | -0.5 |
| C61 | 1.98015 | -1.46732 | -0.5 |
| C62 | 1.48574 | -1.79177 | -0.5 |
| C63 | 1.48992 | -1.83447 | -0.5 |
| C64 | 1.45672 | -1.85771 | -0.5 |
| C65 | 1.45994 | -1.8985 | -0.5 |
| C66 | 1.49649 | -1.91663 | -0.5 |
| C67 | 1.52974 | -1.89307 | -0.5 |
| C68 | 1.52651 | -1.8523 | -0.5 |
| C69 | 1.4999 | -1.95986 | -0.5 |
| C70 | 1.46629 | -1.98348 | -0.5 |
| C71 | 1.5368 | -1.97792 | -0.5 |
| C72 | 1.51638 | -1.16769 | -0.5 |
| C73 | 1.54959 | -1.14446 | -0.5 |
| C74 | 1.54636 | -1.10367 | -0.5 |
| C75 | 1.50982 | -1.08553 | -0.5 |
| C76 | 1.47656 | -1.1091 | -0.5 |
| C77 | 1.4798 | -1.14987 | -0.5 |
| C78 | 1.50641 | -1.0423 | -0.5 |
| C79 | 1.54002 | -1.01868 | -0.5 |
| C80 | 1.46951 | -1.02425 | -0.5 |
| C81 | 1.16979 | -1.4886 | -0.5 |
| C82 | 1.14666 | -1.45528 | -0.5 |
| C83 | 1.10587 | -1.45833 | -0.5 |
| C84 | 1.08762 | -1.49481 | -0.5 |
| C85 | 1.11107 | -1.52818 | -0.5 |
| C86 | 1.15185 | -1.52512 | -0.5 |
| C87 | 1.04438 | -1.49802 | -0.5 |
| C88 | 1.02092 | -1.46429 | -0.5 |
| C89 | 1.02616 | -1.53484 | -0.5 |
| C90 | 1.52057 | -1.21039 | -0.5 |
| N91 | 1.23519 | -1.51388 | -0.5 |
| C92 | 1.21249 | -1.48455 | -0.5 |

**Table S2.** AA stacking model of fractional atomic coordinates for TAPP-TZ-COF.

| TAPP-TZ-COF  Space group: *P*1–triclinic  a=34.7002 Å, b=35.4230 Å, c=3.6972 Å  α=90.0000°, β=90.0000°, γ=90.0000° | | | |
| --- | --- | --- | --- |
|  |  |  |  |
| Atom | x | y | z |
| C1 | 1.46284 | -1.30621 | -0.55943 |
| C2 | 1.46561 | -1.34541 | -0.55918 |
| C3 | 1.49747 | -1.36371 | -0.70211 |
| C4 | 1.5265 | -1.34138 | -0.84743 |
| C5 | 1.52405 | -1.30205 | -0.84357 |
| C6 | 1.49212 | -1.28426 | -0.69796 |
| C7 | 1.50061 | -1.40593 | -0.67908 |
| C8 | 1.4682 | -1.42781 | -0.75163 |
| C9 | 1.43575 | -1.41289 | -0.90111 |
| C10 | 1.41 | -1.44147 | -0.91074 |
| C11 | 1.42774 | -1.47274 | -0.76645 |
| N12 | 1.46383 | -1.46486 | -0.67481 |
| C13 | 1.4091 | -1.50747 | -0.71117 |
| C14 | 1.42846 | -1.54063 | -0.60962 |
| C15 | 1.3668 | -1.50854 | -0.74537 |
| C16 | 1.34497 | -1.47897 | -0.60346 |
| C17 | 1.30569 | -1.48019 | -0.61368 |
| C18 | 1.28715 | -1.51147 | -0.7624 |
| C19 | 1.30849 | -1.54095 | -0.90701 |
| C20 | 1.34789 | -1.53938 | -0.90069 |
| C21 | 1.41047 | -1.57758 | -0.58459 |
| C22 | 1.43688 | -1.60164 | -0.49405 |
| C23 | 1.47164 | -1.58001 | -0.452 |
| N24 | 1.46555 | -1.54311 | -0.52599 |
| C25 | 1.50622 | -1.59614 | -0.34244 |
| C26 | 1.53866 | -1.57423 | -0.27038 |
| C27 | 1.57112 | -1.5891 | -0.11933 |
| C28 | 1.59694 | -1.56058 | -0.11225 |
| C29 | 1.57925 | -1.52936 | -0.25907 |
| N30 | 1.54311 | -1.53725 | -0.35066 |
| C31 | 1.5979 | -1.49463 | -0.31575 |
| C32 | 1.57853 | -1.4615 | -0.41826 |
| C33 | 1.59645 | -1.42455 | -0.44268 |
| C34 | 1.56995 | -1.40045 | -0.53032 |
| C35 | 1.53524 | -1.42208 | -0.57219 |
| N36 | 1.5414 | -1.459 | -0.50044 |
| C37 | 1.64017 | -1.49342 | -0.27868 |
| C38 | 1.50922 | -1.63833 | -0.31502 |
| C39 | 1.47998 | -1.66038 | -0.16955 |
| C40 | 1.48234 | -1.69971 | -0.16637 |
| C41 | 1.51443 | -1.7178 | -0.30519 |
| C42 | 1.54393 | -1.69612 | -0.44425 |
| C43 | 1.54124 | -1.65689 | -0.45138 |
| C44 | 1.66225 | -1.52291 | -0.41845 |
| C45 | 1.70152 | -1.52161 | -0.4018 |
| C46 | 1.71977 | -1.49037 | -0.24885 |
| C47 | 1.69817 | -1.4609 | -0.10843 |
| C48 | 1.65879 | -1.46254 | -0.12098 |
| N49 | 1.48911 | -1.24414 | -0.67595 |
| N50 | 1.76006 | -1.48745 | -0.24477 |
| N51 | 1.51741 | -1.758 | -0.31946 |
| C52 | 1.78183 | -1.51403 | -0.36359 |
| C53 | 1.82295 | -1.51008 | -0.36946 |
| C54 | 1.84478 | -1.54067 | -0.48172 |
| C55 | 1.884 | -1.53751 | -0.49856 |
| C56 | 1.90183 | -1.50364 | -0.40445 |
| C57 | 1.87983 | -1.473 | -0.29199 |
| C58 | 1.84058 | -1.47617 | -0.27422 |
| C59 | 1.94319 | -1.50038 | -0.42917 |
| N60 | 1.96492 | -1.53017 | -0.52853 |
| S61 | 1.96702 | -1.45845 | -0.34274 |
| C62 | 1.49027 | -1.78062 | -0.22972 |
| C63 | 1.49476 | -1.82157 | -0.25971 |
| C64 | 1.46383 | -1.84495 | -0.19217 |
| C65 | 1.4671 | -1.88399 | -0.23389 |
| C66 | 1.50141 | -1.90004 | -0.34302 |
| C67 | 1.53244 | -1.87652 | -0.40662 |
| C68 | 1.52916 | -1.83753 | -0.36595 |
| C69 | 1.50448 | -1.94103 | -0.39879 |
| N70 | 1.47414 | -1.96408 | -0.36135 |
| C71 | 1.48514 | -1.9992 | -0.43569 |
| C72 | 1.52122 | -2.00306 | -0.52402 |
| S73 | 1.5465 | -1.96266 | -0.52451 |
| C74 | 1.51165 | -1.18038 | -0.72366 |
| C75 | 1.54256 | -1.15683 | -0.78747 |
| C76 | 1.53924 | -1.11791 | -0.73924 |
| C77 | 1.50491 | -1.10209 | -0.62738 |
| C78 | 1.47389 | -1.12575 | -0.56705 |
| C79 | 1.47723 | -1.16464 | -0.61427 |
| C80 | 1.50185 | -1.06118 | -0.56577 |
| N81 | 1.53217 | -1.03812 | -0.6014 |
| S82 | 1.45984 | -1.0396 | -0.43747 |
| C83 | 1.18423 | -1.49187 | -0.62234 |
| C84 | 1.16255 | -1.46129 | -0.50708 |
| C85 | 1.12334 | -1.46443 | -0.4853 |
| C86 | 1.10539 | -1.49828 | -0.57717 |
| C87 | 1.12723 | -1.52888 | -0.69375 |
| C88 | 1.16644 | -1.52572 | -0.7167 |
| C89 | 1.06407 | -1.50157 | -0.54893 |
| N90 | 1.04238 | -1.47178 | -0.44969 |
| C91 | 1.00649 | -1.48307 | -0.4447 |
| C92 | 1.0008 | -1.51887 | -0.53321 |
| S93 | 1.04021 | -1.54345 | -0.636 |
| C94 | 1.51617 | -1.22127 | -0.76197 |
| N95 | 1.24686 | -1.51438 | -0.75971 |
| C96 | 1.22535 | -1.48799 | -0.63341 |

**Table S3.** AA stacking model of fractional atomic coordinates for TAPP-TZ-OMe-COF.

| TAPP-TZ-OMe-COF  Space group: *P*1-triclinic  a=35.9401 Å, b=35.5463 Å, c=3.7457 Å  α=90.0000°, β=72.9059°, γ=90.0000° | | | |
| --- | --- | --- | --- |
|  |  |  |  |
| Atom | x | y | z |
| C1 | 1.45997 | -1.30936 | 0.01028 |
| C2 | 1.46202 | -1.34861 | -0.01519 |
| C3 | 1.49445 | -1.36648 | -0.26156 |
| C4 | 1.52501 | -1.34403 | -0.47889 |
| C5 | 1.5232 | -1.30483 | -0.45148 |
| C6 | 1.49045 | -1.28724 | -0.20779 |
| C7 | 1.49613 | -1.40847 | -0.30697 |
| C8 | 1.46334 | -1.42756 | -0.35505 |
| C9 | 1.42826 | -1.40984 | -0.32661 |
| C10 | 1.40369 | -1.43689 | -0.3852 |
| C11 | 1.42476 | -1.47006 | -0.45319 |
| N12 | 1.46157 | -1.46463 | -0.43383 |
| C13 | 1.40908 | -1.50463 | -0.53192 |
| C14 | 1.43193 | -1.53763 | -0.62413 |
| C15 | 1.36647 | -1.50675 | -0.51315 |
| C16 | 1.34781 | -1.47692 | -0.6386 |
| C17 | 1.30819 | -1.47865 | -0.61306 |
| C18 | 1.28619 | -1.51064 | -0.4629 |
| C19 | 1.30433 | -1.54055 | -0.3377 |
| C20 | 1.3439 | -1.53845 | -0.35931 |
| C21 | 1.41748 | -1.57269 | -0.73984 |
| C22 | 1.44547 | -1.59764 | -0.78343 |
| C23 | 1.47787 | -1.57844 | -0.69747 |
| N24 | 1.46897 | -1.54194 | -0.60811 |
| C25 | 1.51273 | -1.59691 | -0.69783 |
| C26 | 1.5457 | -1.57776 | -0.65468 |
| C27 | 1.58087 | -1.59542 | -0.68524 |
| C28 | 1.60561 | -1.56823 | -0.63206 |
| C29 | 1.58451 | -1.53508 | -0.56442 |
| N30 | 1.54756 | -1.54063 | -0.57929 |
| C31 | 1.6002 | -1.50044 | -0.48839 |
| C32 | 1.57721 | -1.46758 | -0.39082 |
| C33 | 1.5916 | -1.43264 | -0.27193 |
| C34 | 1.56348 | -1.40777 | -0.22377 |
| C35 | 1.53108 | -1.4269 | -0.31039 |
| N36 | 1.54009 | -1.46329 | -0.40435 |
| C37 | 1.64298 | -1.49795 | -0.51642 |
| C38 | 1.51397 | -1.63899 | -0.73255 |
| C39 | 1.48309 | -1.66066 | -0.50919 |
| C40 | 1.48433 | -1.69989 | -0.52495 |
| C41 | 1.5168 | -1.71839 | -0.76283 |
| C42 | 1.54765 | -1.69707 | -0.98722 |
| C43 | 1.54617 | -1.65772 | -0.97353 |
| C44 | 1.6624 | -1.52737 | -0.39342 |
| C45 | 1.70224 | -1.52505 | -0.42787 |
| C46 | 1.72357 | -1.4929 | -0.58458 |
| C47 | 1.70463 | -1.46347 | -0.70746 |
| C48 | 1.66491 | -1.46614 | -0.677 |
| N49 | 1.48699 | -1.24709 | -0.18485 |
| N50 | 1.76408 | -1.48891 | -0.61217 |
| N51 | 1.5196 | -1.75863 | -0.77127 |
| C52 | 1.78458 | -1.51534 | -0.52182 |
| C53 | 1.8259 | -1.5095 | -0.54681 |
| C54 | 1.8481 | -1.53916 | -0.46886 |
| C55 | 1.8874 | -1.53248 | -0.49023 |
| C56 | 1.90492 | -1.49712 | -0.58609 |
| C57 | 1.88245 | -1.46737 | -0.66858 |
| C58 | 1.84313 | -1.47422 | -0.6463 |
| C59 | 1.94638 | -1.49305 | -0.59055 |
| N60 | 1.96808 | -1.52391 | -0.56855 |
| S61 | 1.97004 | -1.44993 | -0.60352 |
| C62 | 1.49185 | -1.78007 | -0.57463 |
| C63 | 1.4948 | -1.82139 | -0.57065 |
| C64 | 1.46216 | -1.84073 | -0.35248 |
| C65 | 1.46184 | -1.88016 | -0.3168 |
| C66 | 1.49572 | -1.90059 | -0.50619 |
| C67 | 1.52842 | -1.88103 | -0.72557 |
| C68 | 1.52859 | -1.84161 | -0.76321 |
| C69 | 1.4982 | -1.94212 | -0.47756 |
| N70 | 1.46619 | -1.96373 | -0.32568 |
| C71 | 1.47757 | -1.99957 | -0.33545 |
| C72 | 1.5155 | -2.00547 | -0.48393 |
| S73 | 1.54235 | -1.9663 | -0.63472 |
| C74 | 1.50918 | -1.18348 | -0.35388 |
| C75 | 1.53931 | -1.16005 | -0.5603 |
| C76 | 1.53337 | -1.12089 | -0.54386 |
| C77 | 1.49837 | -1.10466 | -0.32703 |
| C78 | 1.46836 | -1.12841 | -0.11251 |
| C79 | 1.47434 | -1.16763 | -0.1321 |
| C80 | 1.49482 | -1.06303 | -0.34079 |
| N81 | 1.52682 | -1.04136 | -0.49438 |
| S82 | 1.45067 | -1.03873 | -0.18922 |
| C83 | 1.18534 | -1.49023 | -0.5314 |
| C84 | 1.16713 | -1.45774 | -0.60693 |
| C85 | 1.12809 | -1.45789 | -0.61471 |
| C86 | 1.10699 | -1.49208 | -0.54693 |
| C87 | 1.12543 | -1.52462 | -0.47143 |
| C88 | 1.16437 | -1.52438 | -0.46087 |
| C89 | 1.06599 | -1.49499 | -0.5571 |
| N90 | 1.04435 | -1.46415 | -0.57872 |
| C91 | 1.00906 | -1.47561 | -0.58381 |
| C92 | 1.00351 | -1.51252 | -0.56648 |
| S93 | 1.04245 | -1.53819 | -0.54198 |
| C94 | 1.51405 | -1.22459 | -0.37559 |
| N95 | 1.24586 | -1.51385 | -0.44586 |
| C96 | 1.22648 | -1.48661 | -0.53841 |
| O97 | 0.83025 | 1.42551 | 0.63091 |
| O98 | 0.89967 | 1.56803 | 0.22257 |
| C99 | 0.87487 | 1.59633 | 0.14632 |
| C100 | 0.85462 | 1.39672 | 0.70541 |
| O101 | 1.18276 | 1.44278 | 0.61418 |
| C102 | 1.15916 | 1.40976 | 0.69168 |
| O103 | 1.11006 | 1.57497 | 0.30527 |
| C104 | 1.13354 | 1.60807 | 0.23486 |
| O105 | 0.56152 | 1.17791 | 0.01599 |
| C106 | 0.59428 | 1.1553 | -0.17096 |
| O107 | 0.42897 | 1.10058 | 0.90884 |
| C108 | 0.39609 | 1.12327 | 1.09093 |
| O109 | 0.57427 | 0.82347 | 0.21981 |
| C110 | 0.60367 | 0.84902 | 0.02017 |
| O111 | 0.43366 | 0.88734 | 1.12029 |
| C112 | 0.4047 | 0.86136 | 1.31978 |

**Table S4.** Comparison of the recovery capacities of reported porous materials.

| Adsorbent | pH | Equilibrium time | Recovery capacity (mg g^−1^) | Recovery rate with the  presence of competing ions | Ref. |
| --- | --- | --- | --- | --- | --- |
| TAPP-TZ-OMe-COF | 3 | 18 h | 4109 | ~100% | **This work** |
| TAPP-TZ-COF | 3 | 22 h | 3548 | > 99.9% |  |
| TAPP-TB-COF | 3 | 22 h | 2766 | > 99.9% |  |
| APPD-TTP-COF | 2-8 | 12 h | 4890 | > 99% | [47] |
| PP-COF | 3.28 | 12 h | 4729 | 99.8% | [34] |
| TpTsc | 5 | 48 h | 4400.23 | 99.69% | [48] |
| Py-COF-N_2_ | 2.33 | 12 h | 4284 | >99% | [49] |
| Furan–COF–2 | 2.33 | 12 h | 3834 | >98% | [50] |
| ECUT-COF-29 | 2 | 1h | 3714 | 99% | [51] |
| JNM-103 | 4 | 24 h | 3687 | 99% | [52] |
| NKCOF-77 | 2 | 12 h | 3225 | > 99.9% | [53] |
| Tp-BTD-AA | 5 | 6 h | 3094.6 | 99% | [54] |
| TPDA-DPTA-COF | 2-10 | 5 d | 3014 | 99.5% | [55] |
| COF-TPTD-DHTA-TAB | 5 | 5 h | 2884 | >98% | [56] |
| BTP-DET | 2 | 250 min | 2580.8 | >97% | [13] |
| MoS_2_-TPTA-1.5 | 5 | 24 h | 2564.8 | 99.8% | [57] |
| TTF-COF | 4 | 4 h | 2440 | > 99% | [30] |
| HATP-COF-2 | 2-10 | 12 h | 2365 | 99.69% | [58] |
| PYTA-PZDH-COF | 7 | 4 h | 2314 | ~100% | [59] |
| PbTu-CTF | 2-5 | 24 h | 2255 | ~100% | [60] |
| Ptriaz-CN-A | 2-10 | 24 h | 2090 | 99.8% | [26] |
| COF-2 | 3 | 8 h | 2081 | >98% | [61] |
| CF-COF | 3 | 95 min | 1895 | >98% | [62] |
| N-PYTA-PATA-COF | 2-10 | 12 h | 1834 | 97.45% | [29] |
| MTpPa-1 | 2 | 24 h | 1737 | 98% | [63] |
| COF-HNU25 | 7 | 4 h | 1725 | 99% | [1] |
| DB18C6-HCP | 1-10 | 12 h | 1667 | 99.8% | [64] |
| im-PYTA-PZDH-COF | 2 | 12 h | 1558 | >98% | [65] |
| isp^2^c-COFox-NH_2_ | 5 | 12 h | 1389 | 99.2% | [66] |
| Ionic-COF-Cl | 2-4.5 | 12 h | 1270.8 | >95% | [28] |
| Fe_3_O_4_@SiO_2_@COF-DMSA | 3 | 12 h | 1116 | 98% | [67] |
| COF-An | 3 | 8 h | 1080.7 | 99% | [68] |
| TY-Hz COF | 3 | 24 h | 1008.3 | ~100% | [69] |
